# Supplementary figures and images for: Dilated cardiomyopathy mutation E525K in human beta-cardiac myosin stabilizes the interacting-heads motif and super-relaxed state of myosin
Source: eLife. 2022 Nov 24;11:e77415. doi: 10.7554/eLife.77415 (PMC9691020; doi:10.7554/eLife.77415)

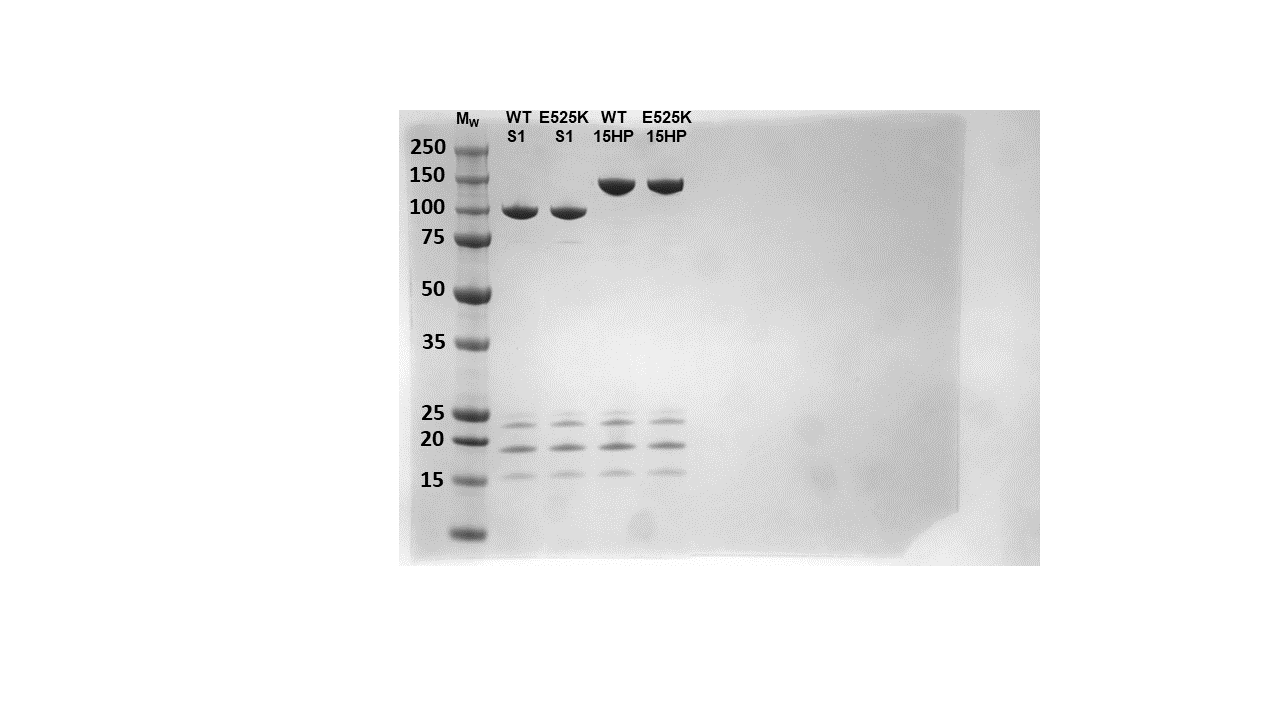

Supplement: Figure 2—figure supplement 1—source data 1. [file elife-77415-fig2-figsupp1-data1.zip › Gel of S1 and 15HP uncropped.png]

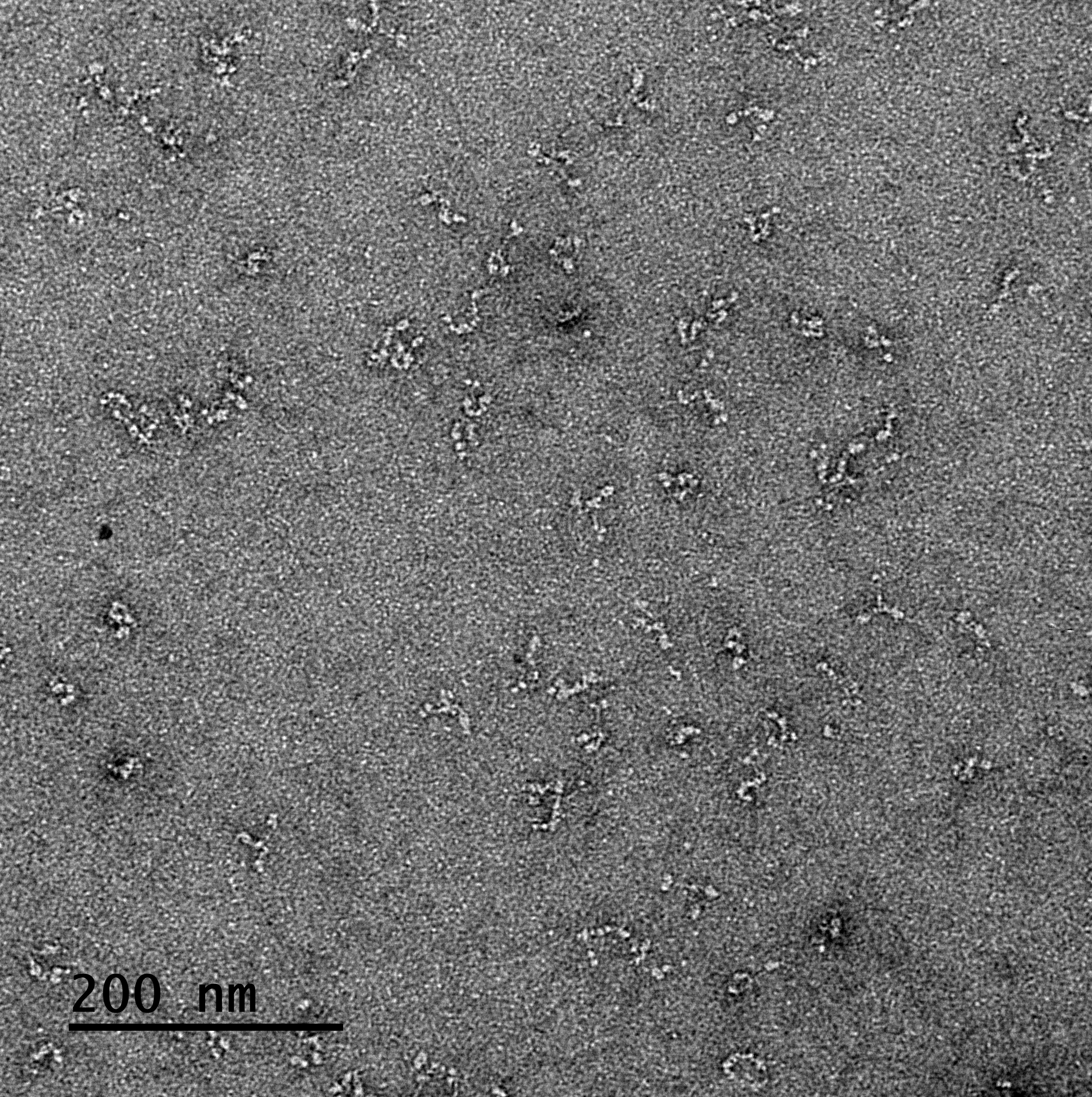

Supplement: Figure 6—source data 1. [file elife-77415-fig6-data1.zip › 1st prep_08-29-2021/E525K_1/E525K_2.tif]
