# Supplementary material for: Dilated cardiomyopathy mutation E525K in human beta-cardiac myosin stabilizes the interacting-heads motif and super-relaxed state of myosin
Source: eLife. 2022 Nov 24;11:e77415. doi: 10.7554/eLife.77415 (PMC9691020; doi:10.7554/eLife.77415)
Supplement: Figure 6—source data 1. [file elife-77415-fig6-data1.zip › 1st prep_08-29-2021/09302021_ %folded particles_15hepHMM RC.pptx]

## Slide 1
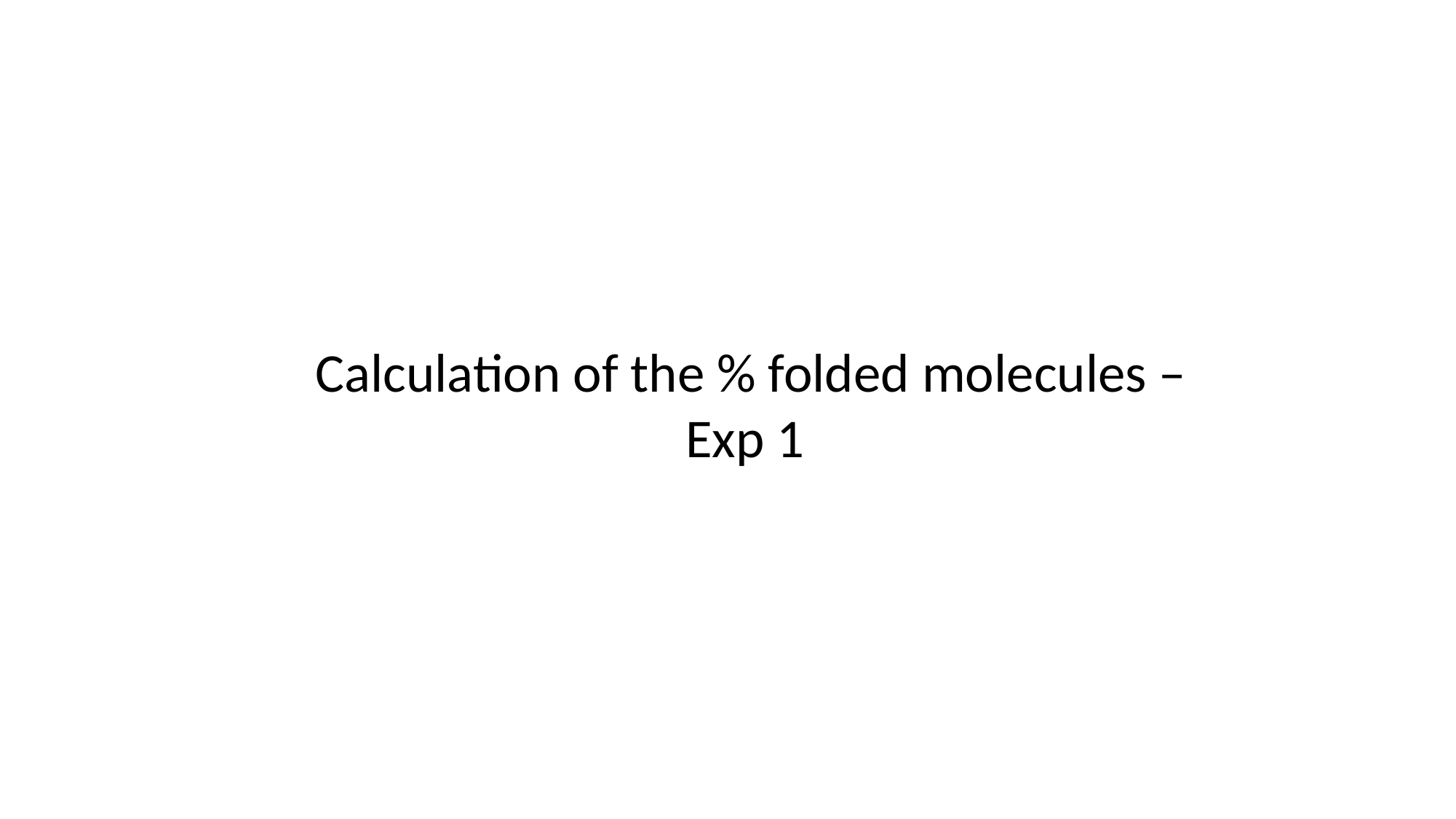

Calculation of the % folded molecules – Exp 1

## Slide 2
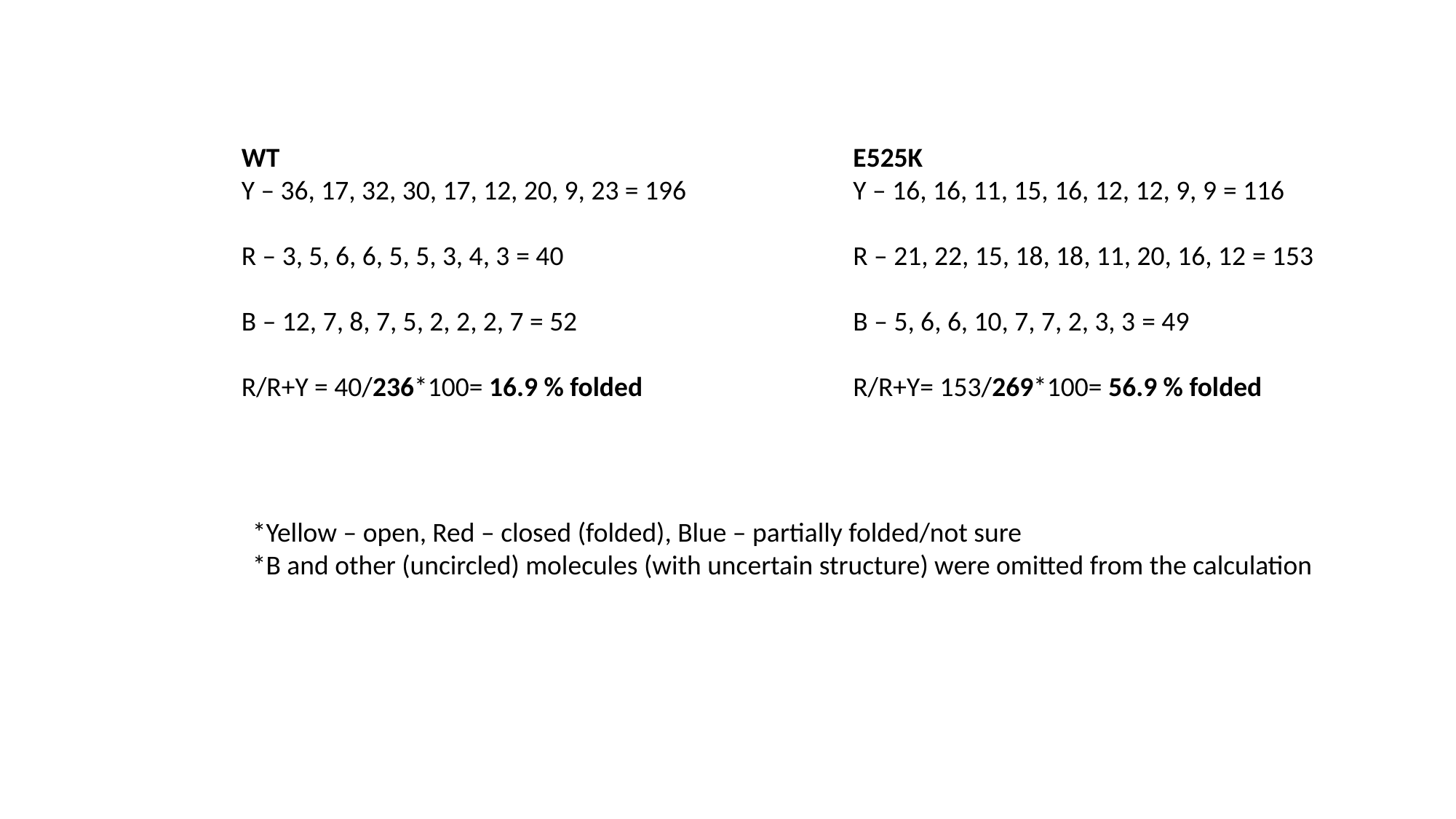

WT
Y – 36, 17, 32, 30, 17, 12, 20, 9, 23 = 196
R – 3, 5, 6, 6, 5, 5, 3, 4, 3 = 40
B – 12, 7, 8, 7, 5, 2, 2, 2, 7 = 52
R/R+Y = 40/236*100= 16.9 % folded
E525K
Y – 16, 16, 11, 15, 16, 12, 12, 9, 9 = 116
R – 21, 22, 15, 18, 18, 11, 20, 16, 12 = 153
B – 5, 6, 6, 10, 7, 7, 2, 3, 3 = 49
R/R+Y= 153/269*100= 56.9 % folded
*Yellow – open, Red – closed (folded), Blue – partially folded/not sure
*B and other (uncircled) molecules (with uncertain structure) were omitted from the calculation

## Slide 3
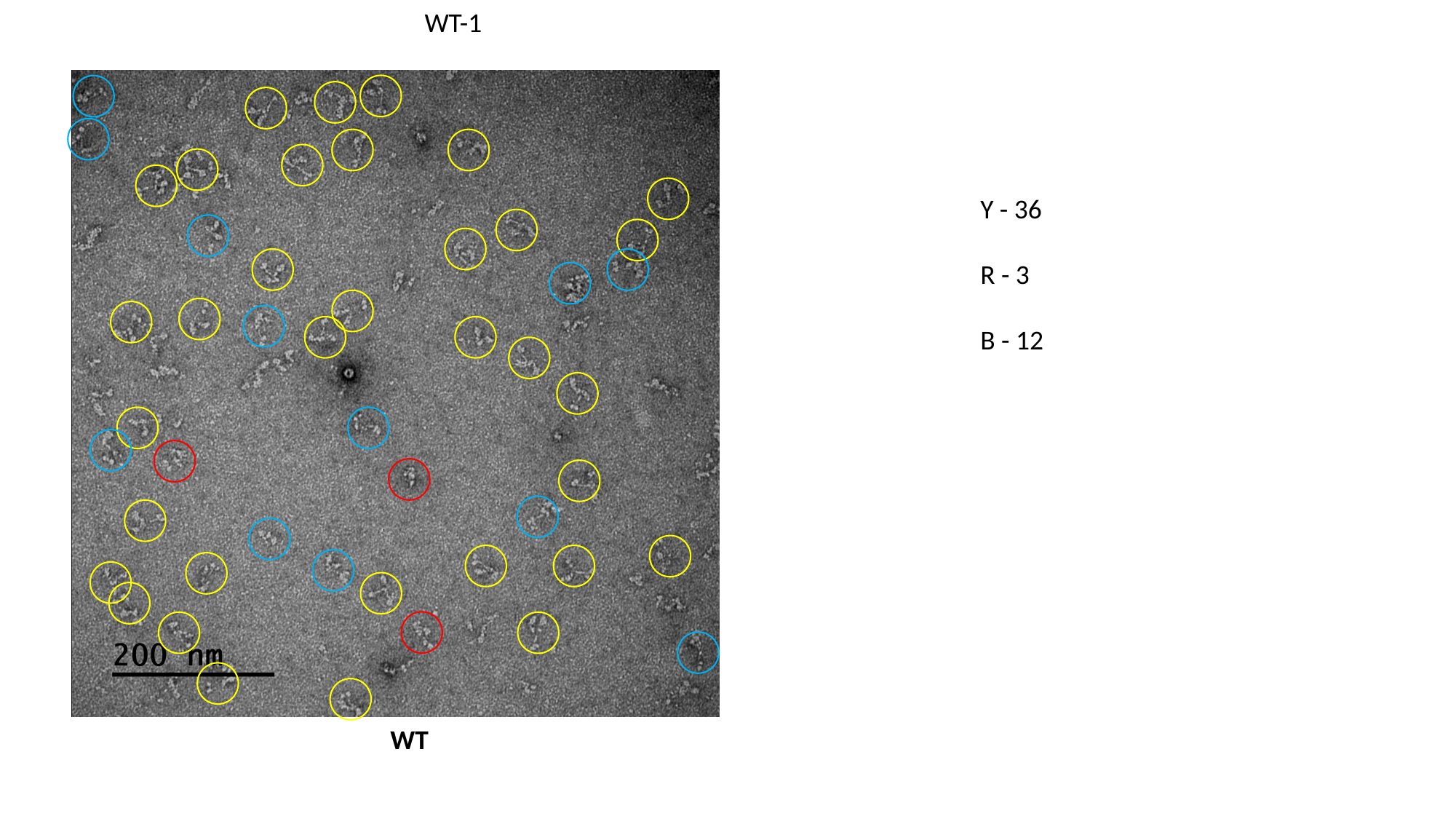

WT-1
Y - 36
R - 3
B - 12
WT

## Slide 4
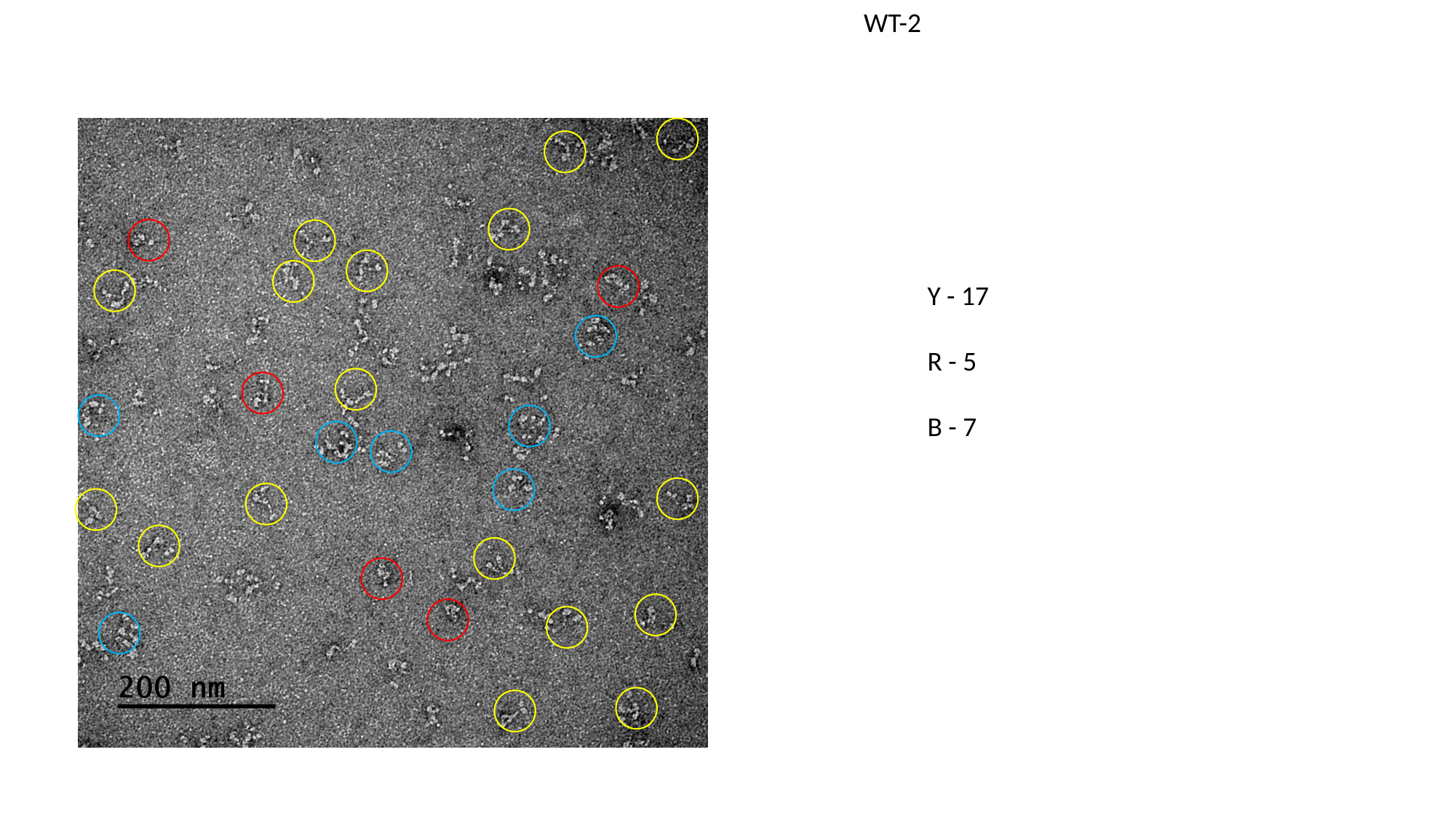

WT-2
Y - 17
R - 5
B - 7

## Slide 5
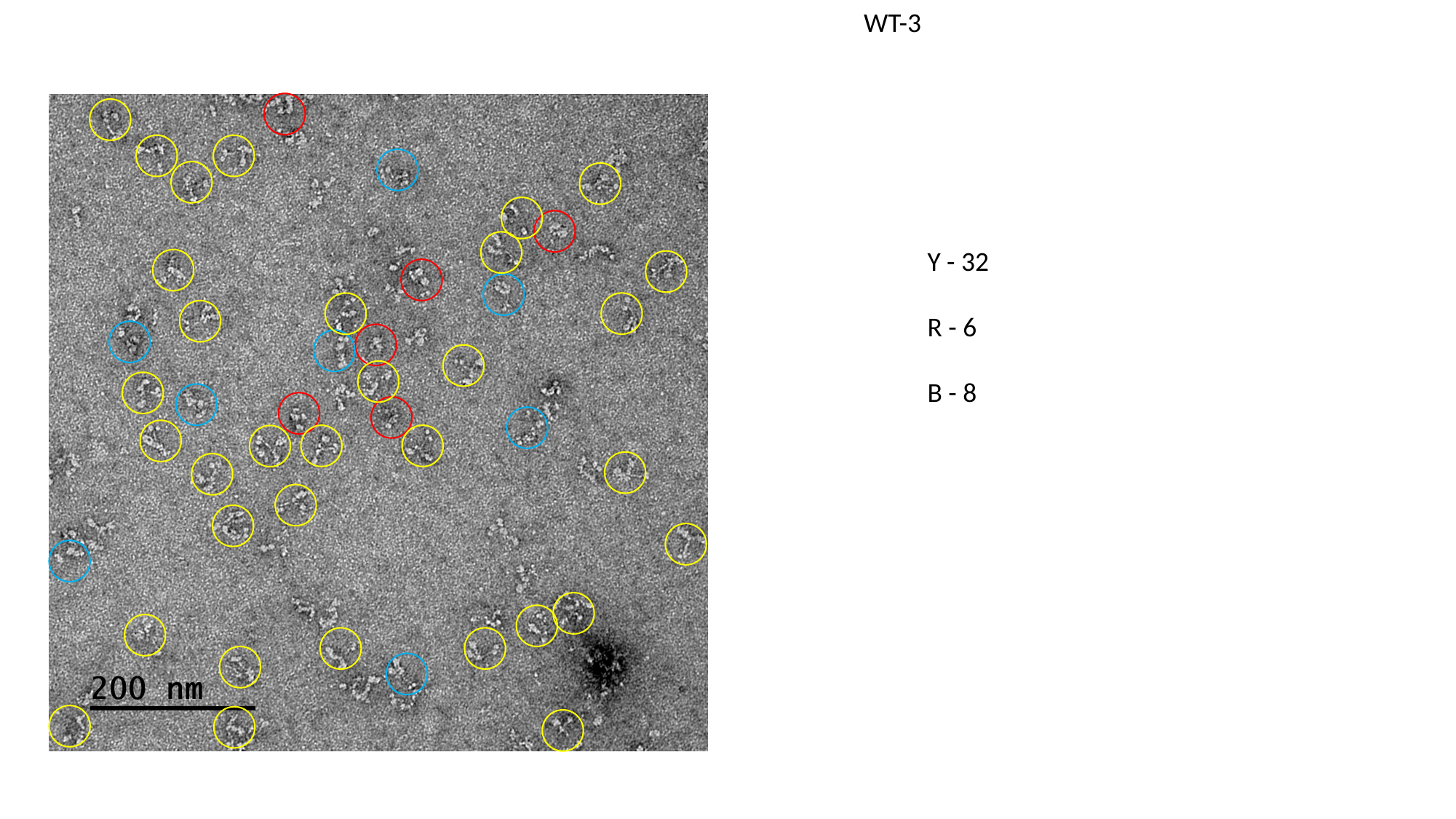

WT-3
Y - 32
R - 6
B - 8

## Slide 6
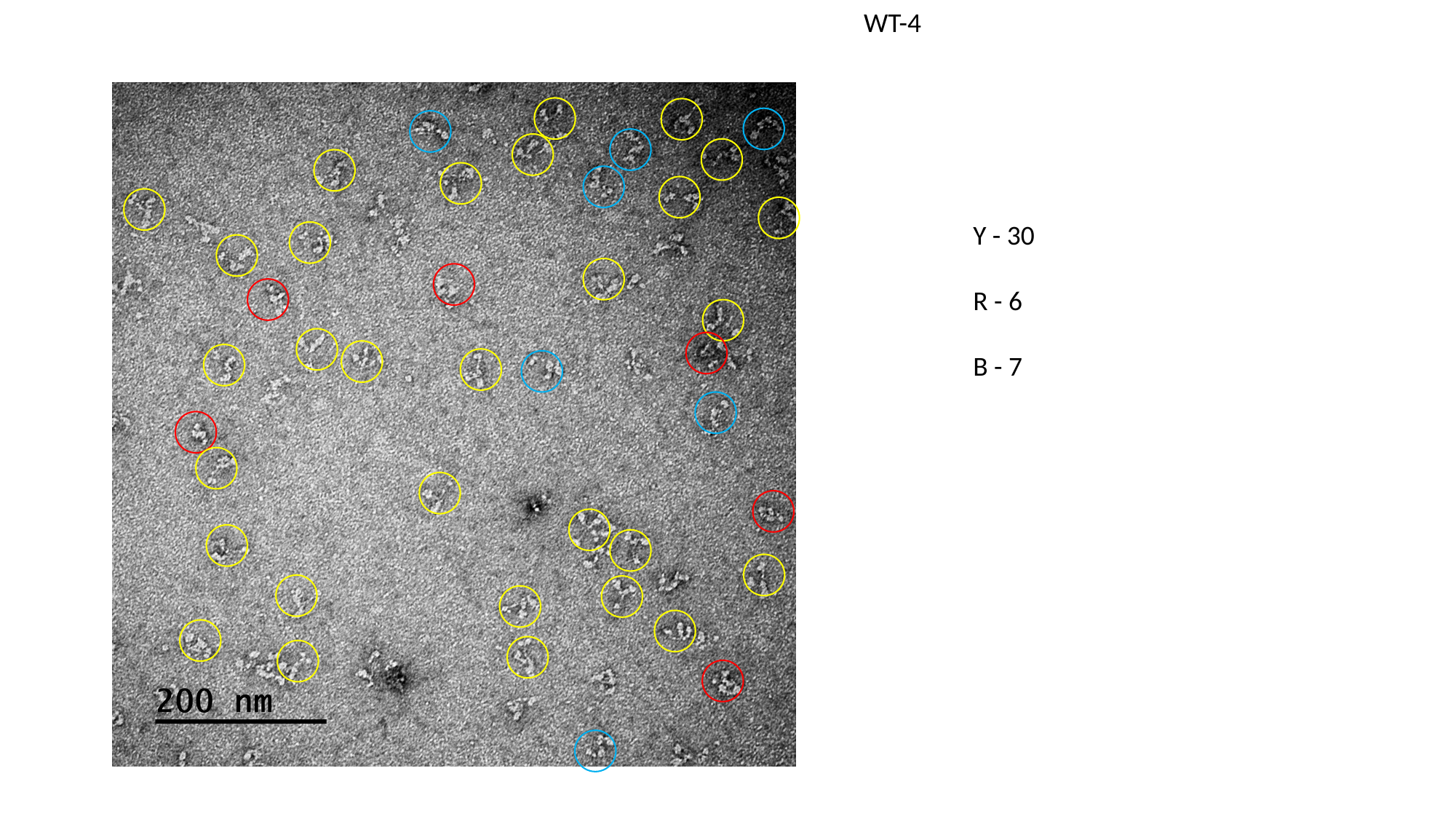

WT-4
Y - 30
R - 6
B - 7

## Slide 7
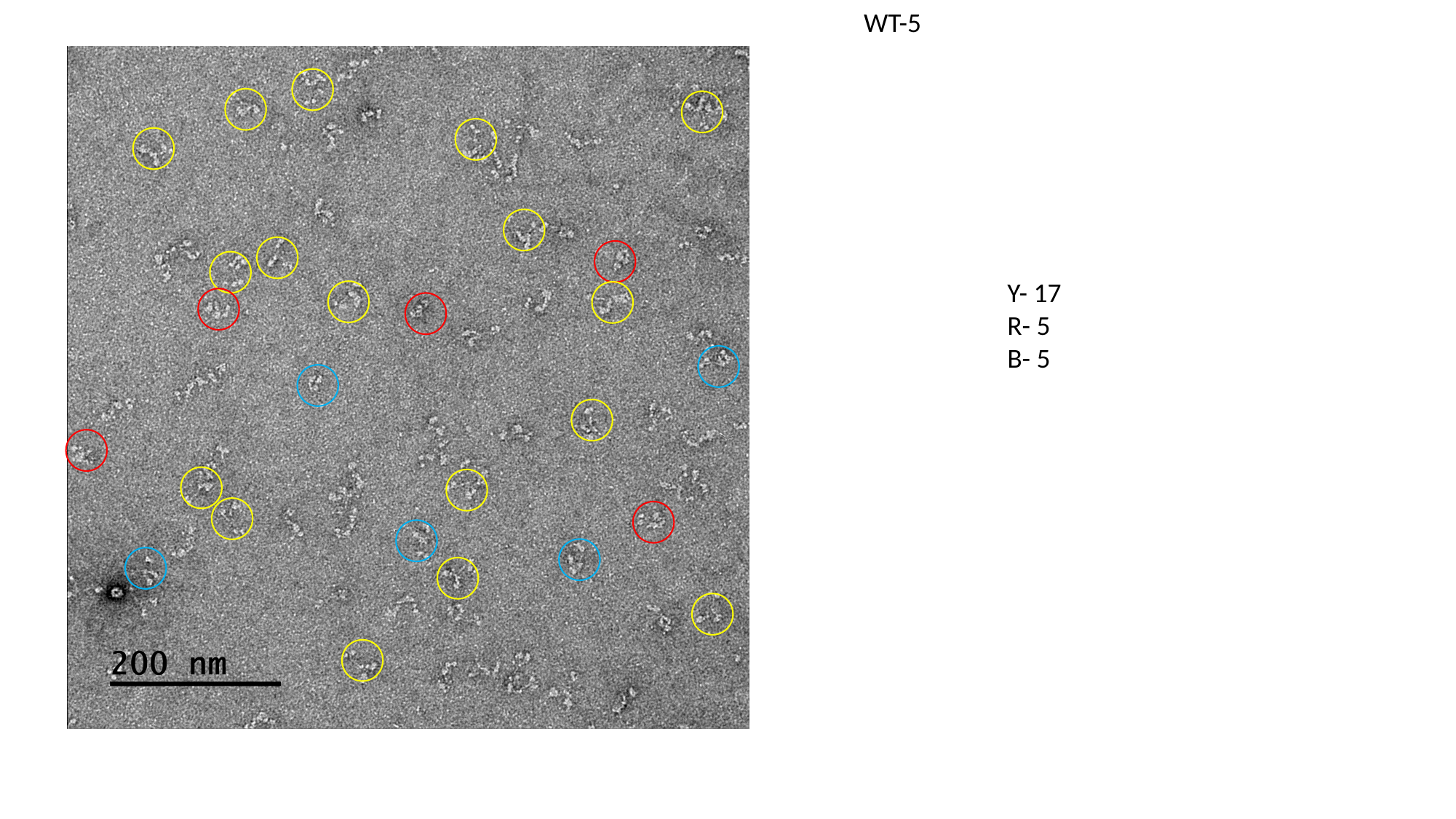

WT-5
Y- 17
R- 5
B- 5

## Slide 8
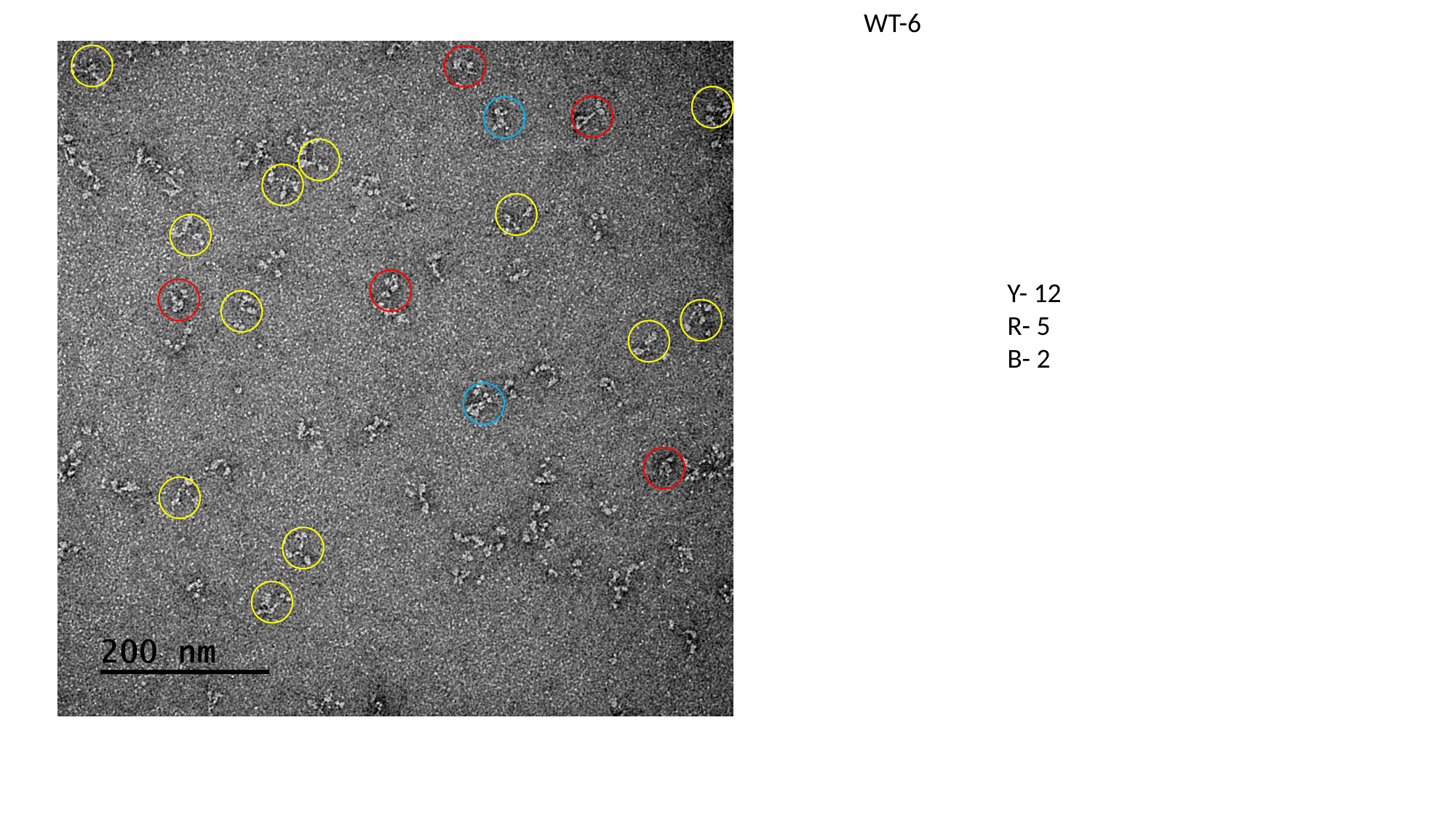

WT-6
Y- 12
R- 5
B- 2

## Slide 9
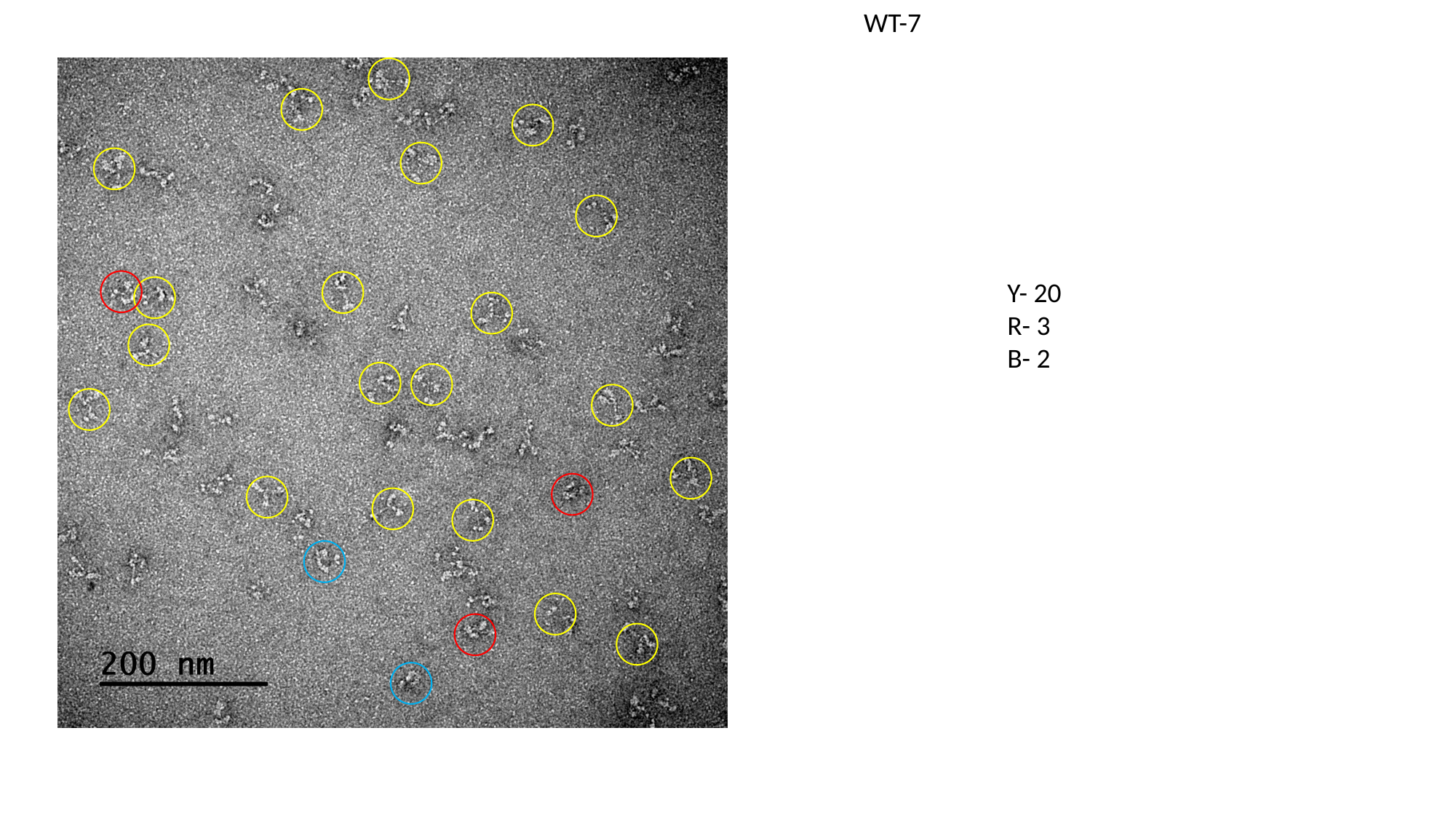

WT-7
Y- 20
R- 3
B- 2

## Slide 10
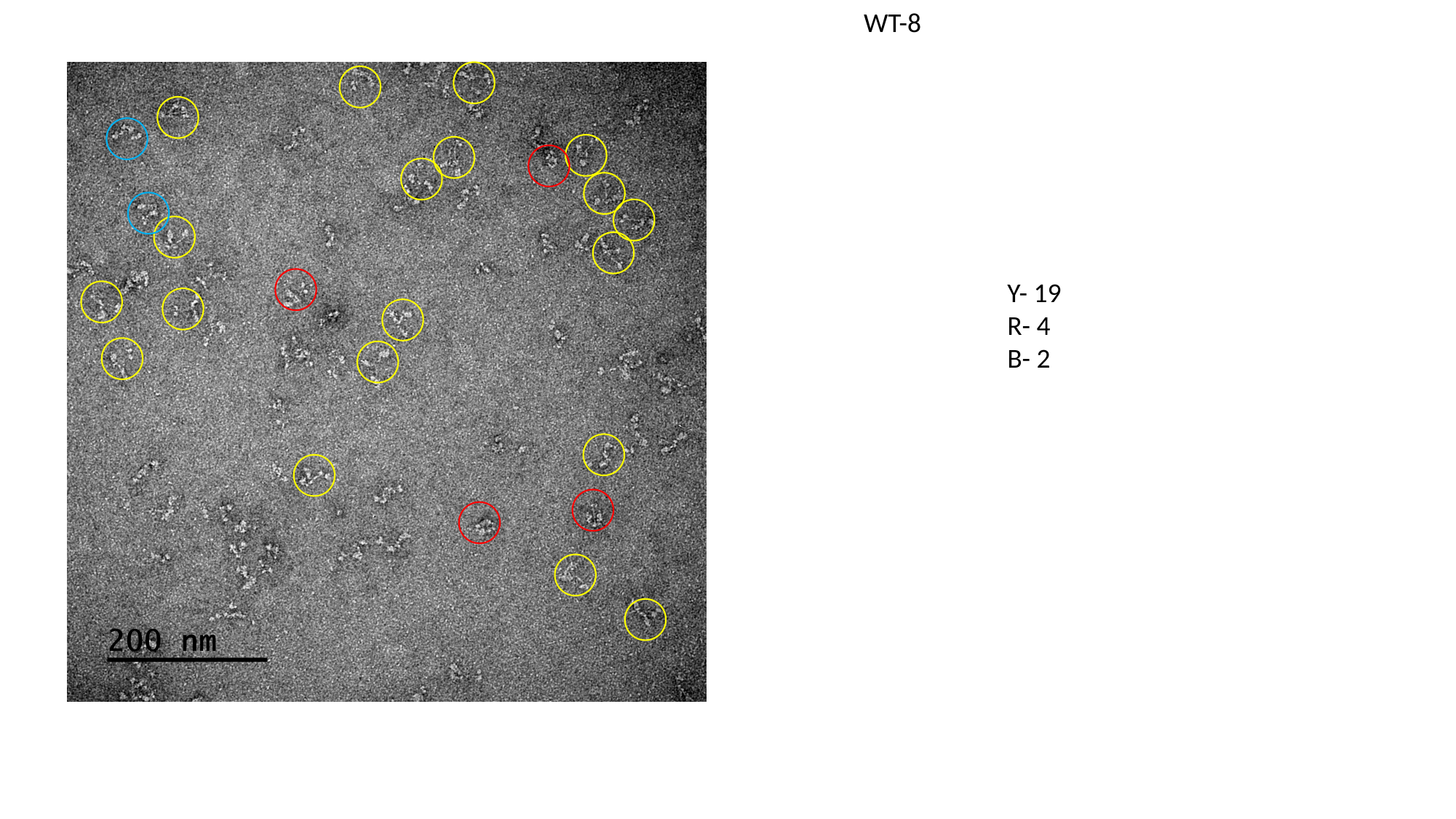

WT-8
Y- 19
R- 4
B- 2

## Slide 11
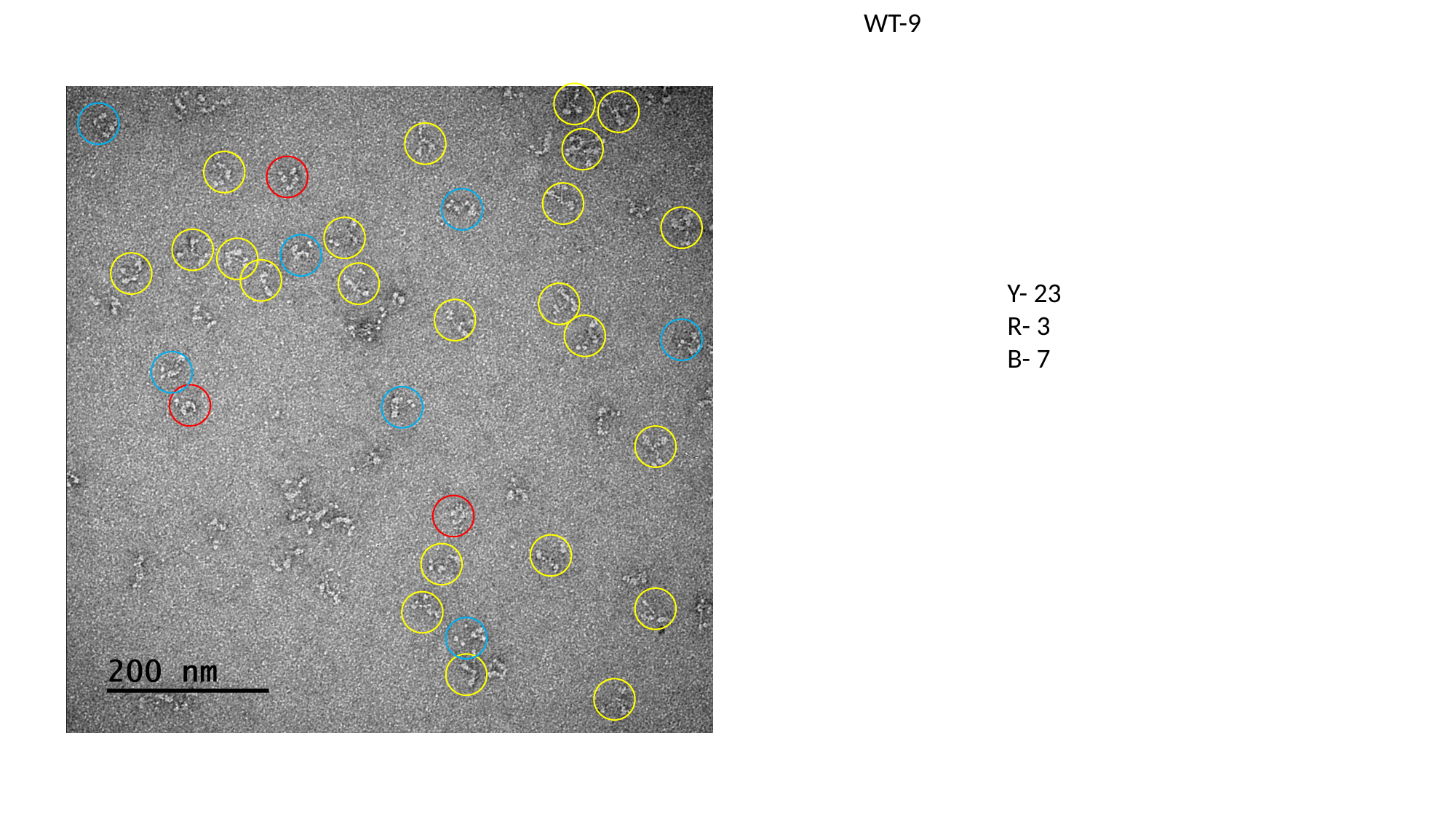

WT-9
Y- 23
R- 3
B- 7

## Slide 12
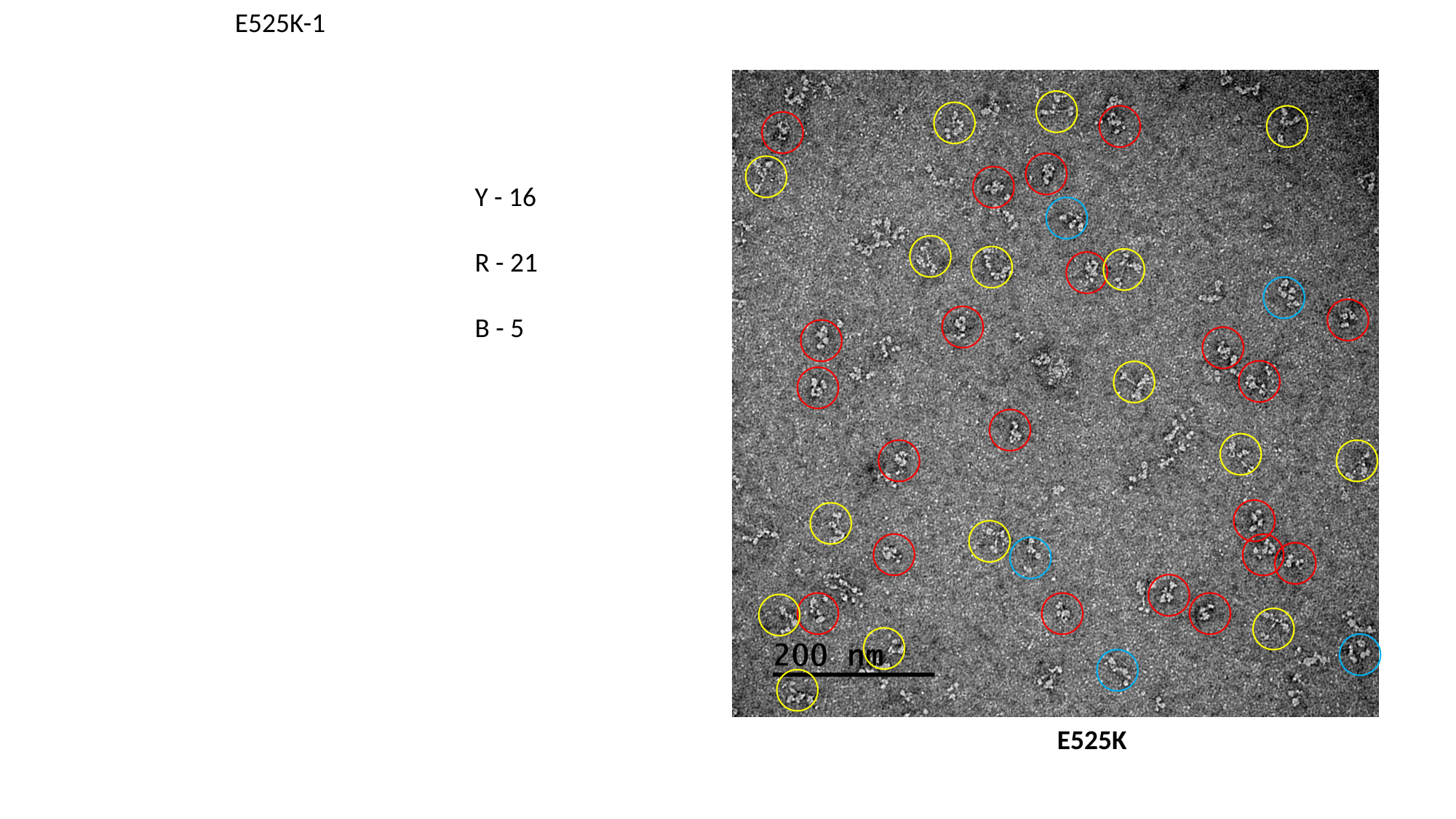

E525K-1
Y - 16
R - 21
B - 5
E525K

## Slide 13
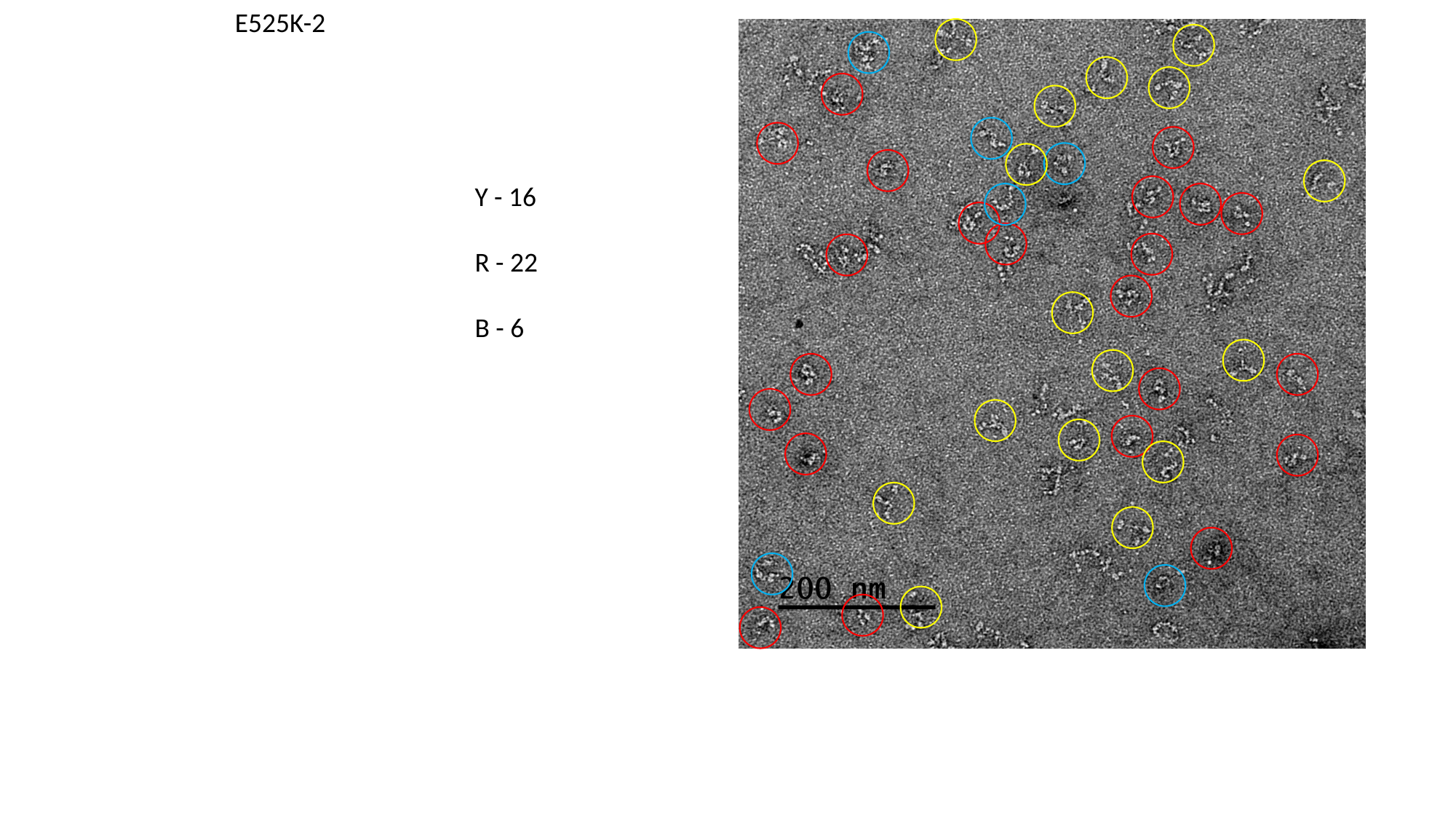

E525K-2
Y - 16
R - 22
B - 6

## Slide 14
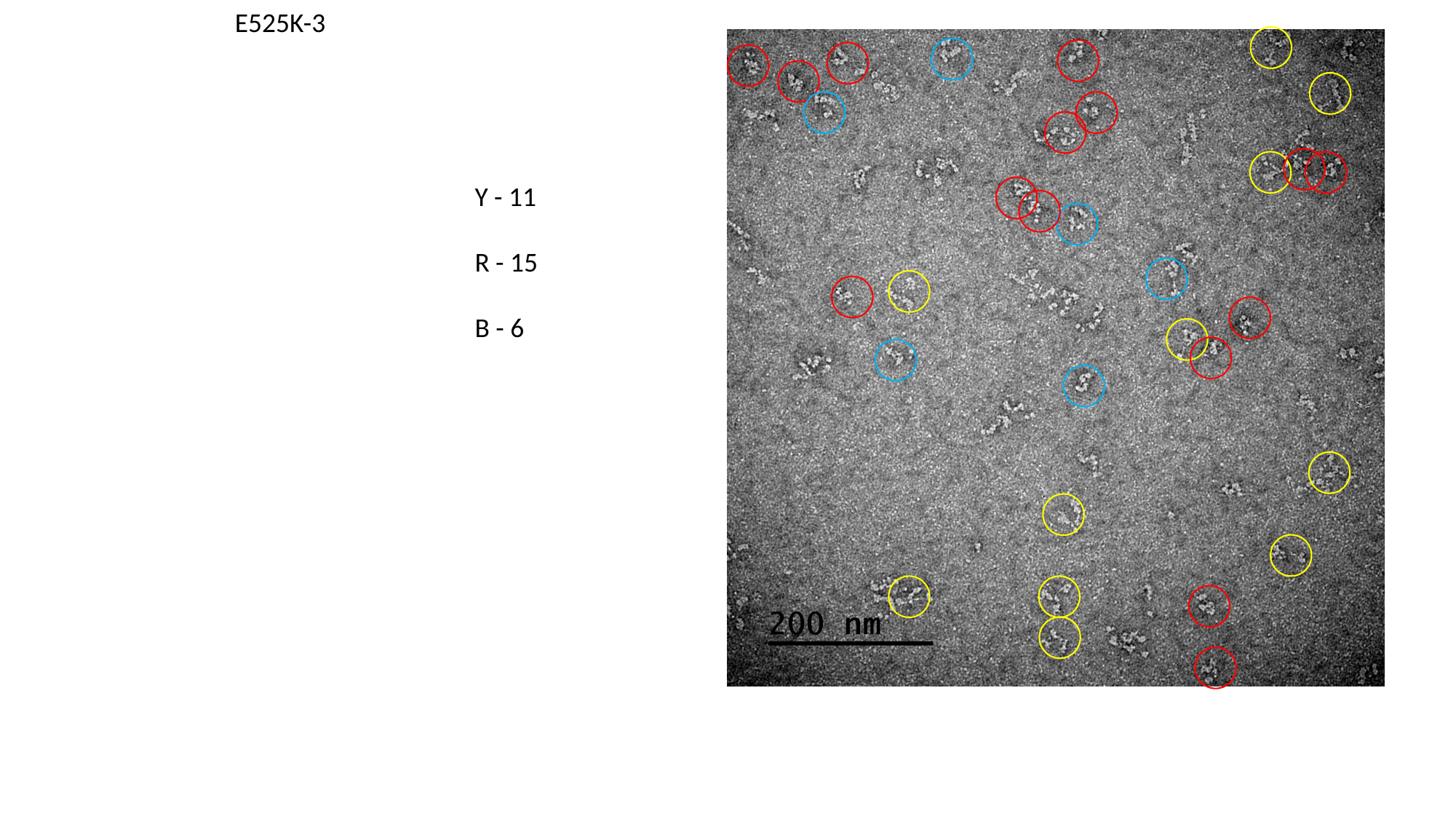

E525K-3
Y - 11
R - 15
B - 6

## Slide 15
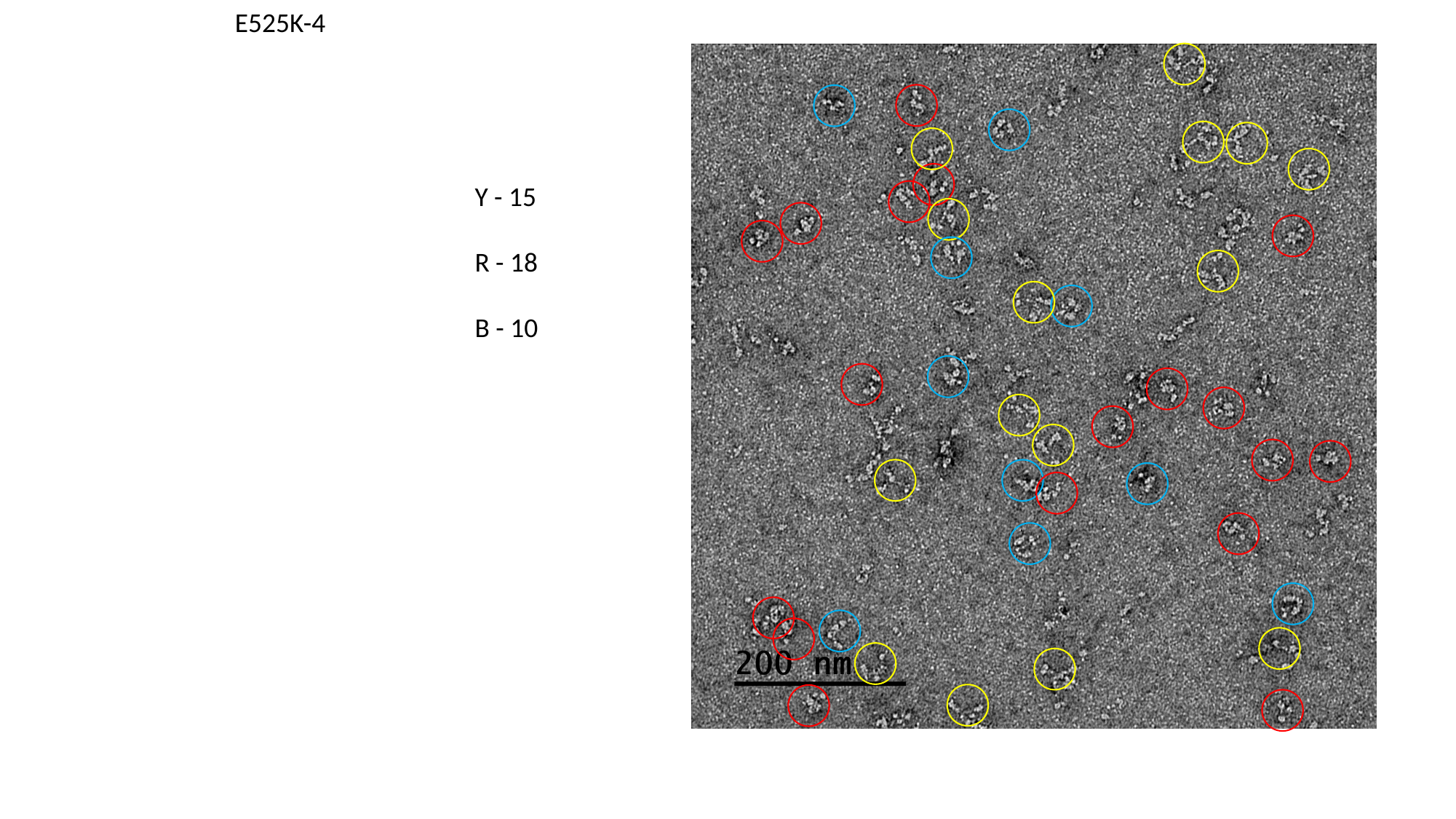

E525K-4
Y - 15
R - 18
B - 10

## Slide 16
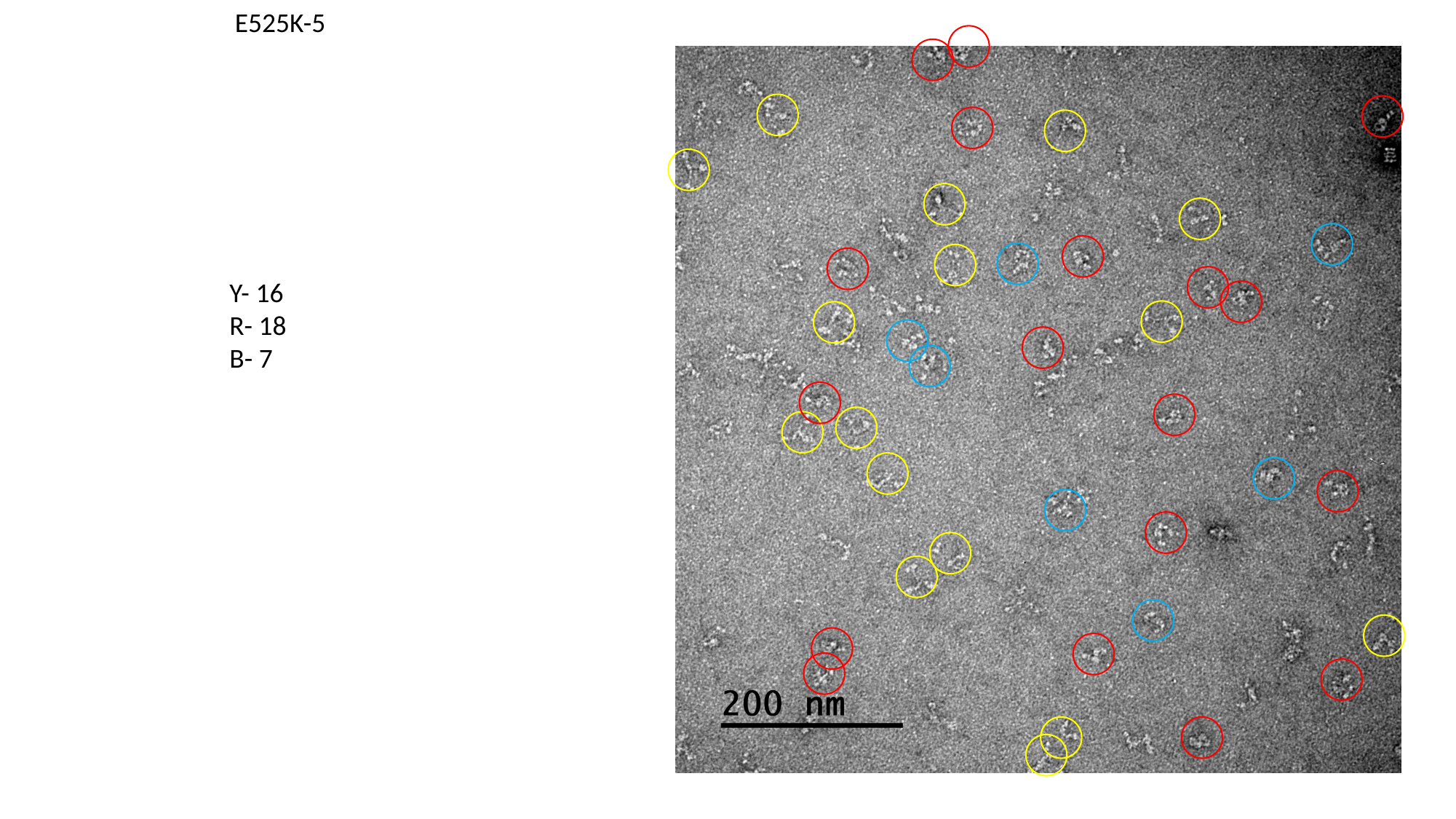

E525K-5
Y- 16
R- 18
B- 7

## Slide 17
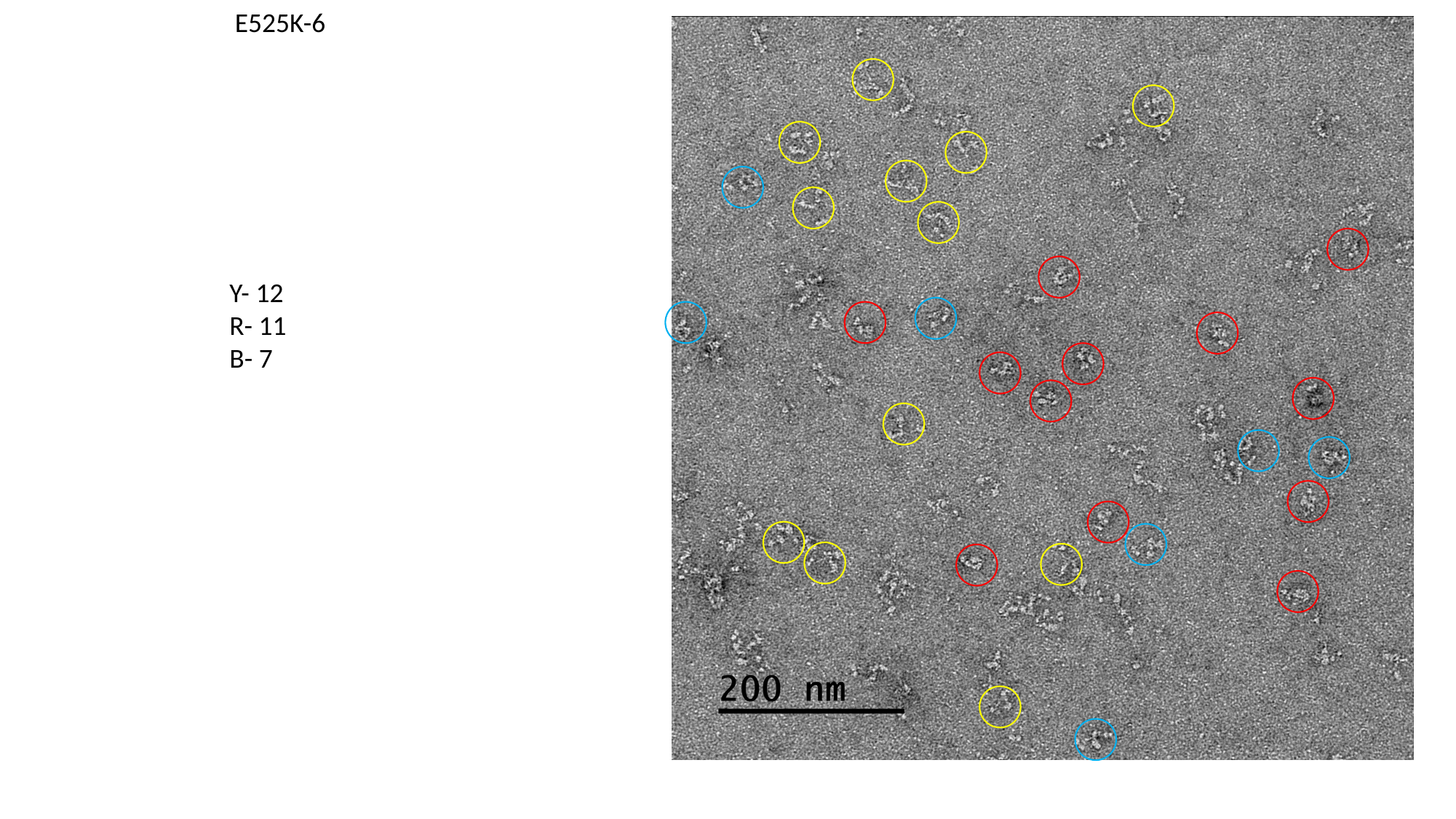

E525K-6
Y- 12
R- 11
B- 7

## Slide 18
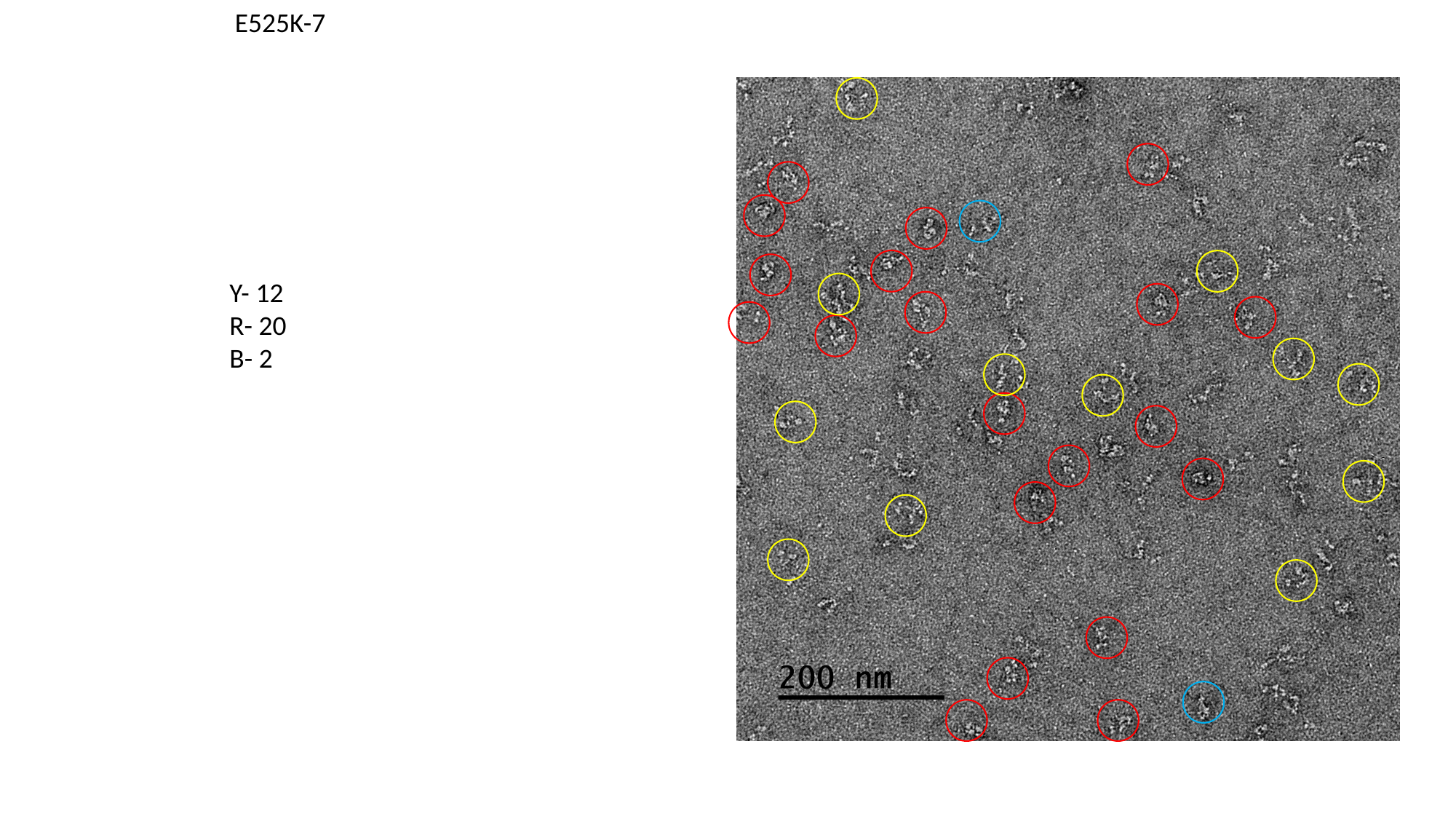

E525K-7
Y- 12
R- 20
B- 2

## Slide 19
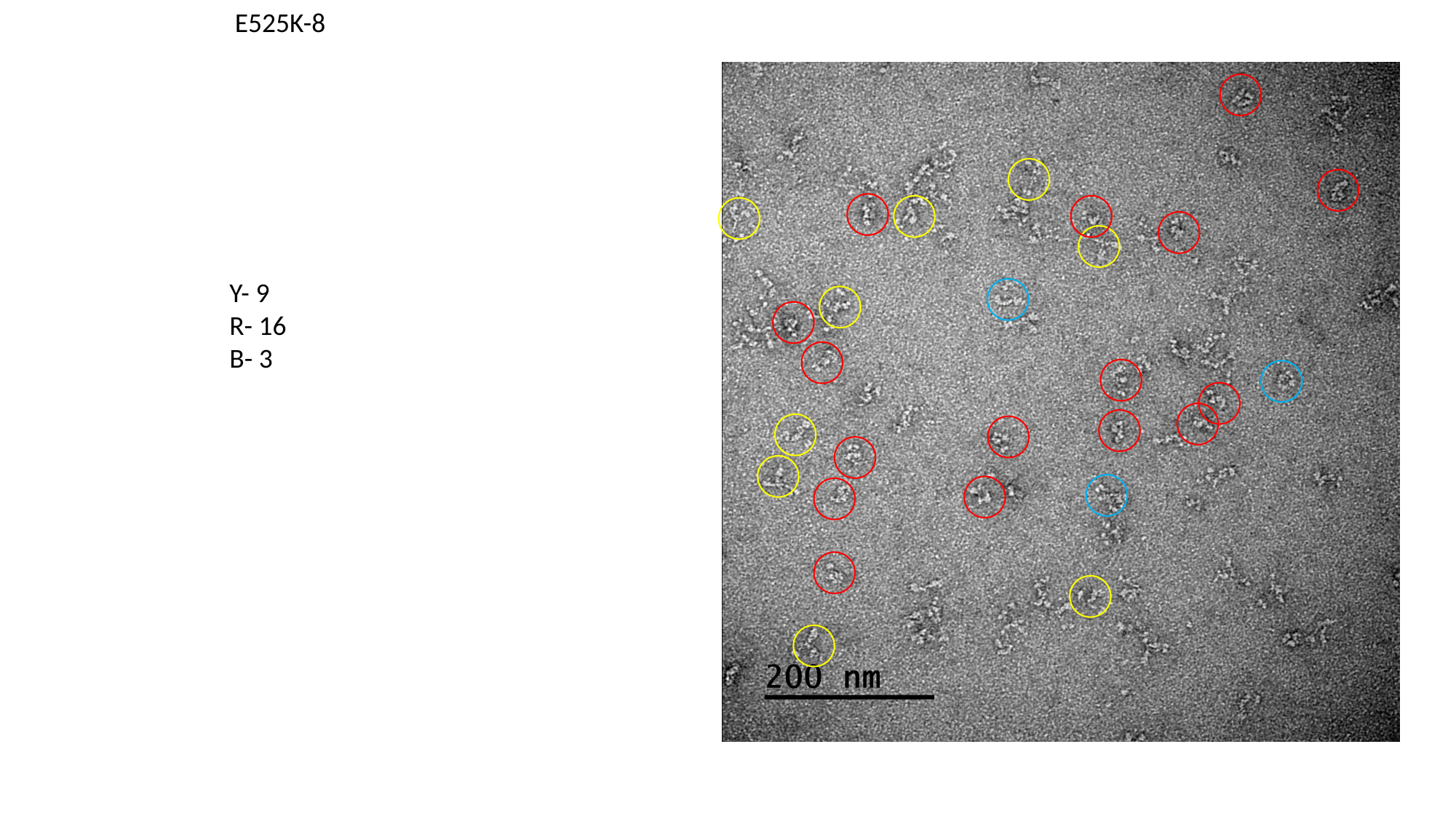

E525K-8
Y- 9
R- 16
B- 3

## Slide 20
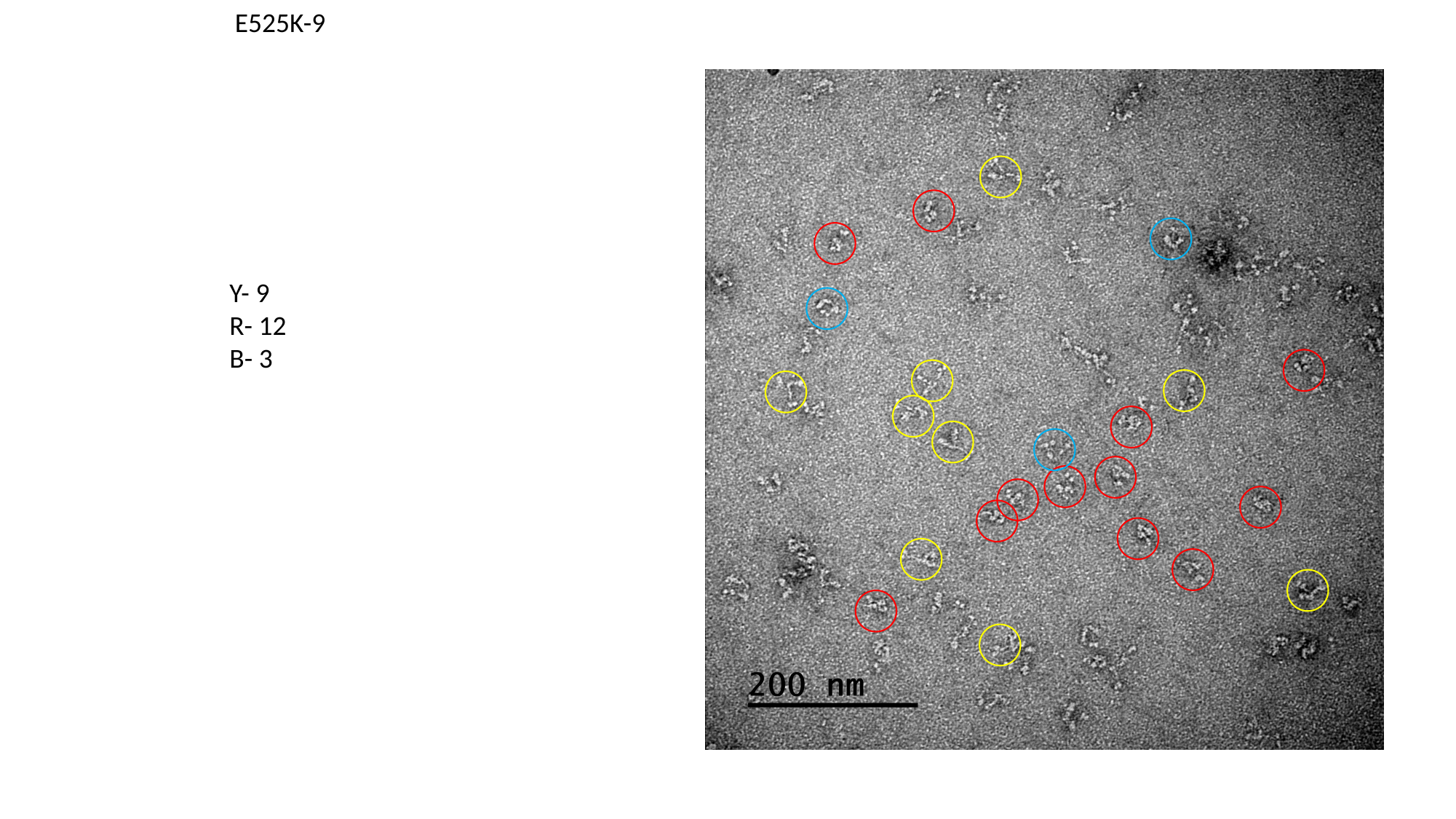

E525K-9
Y- 9
R- 12
B- 3
