# Supplementary material for: Dilated cardiomyopathy mutation E525K in human beta-cardiac myosin stabilizes the interacting-heads motif and super-relaxed state of myosin
Source: eLife. 2022 Nov 24;11:e77415. doi: 10.7554/eLife.77415 (PMC9691020; doi:10.7554/eLife.77415)
Supplement: Figure 6—source data 1. [file elife-77415-fig6-data1.zip › 2nd prep_05-10-2022/E525K/E525K.pptx]

## Slide 1
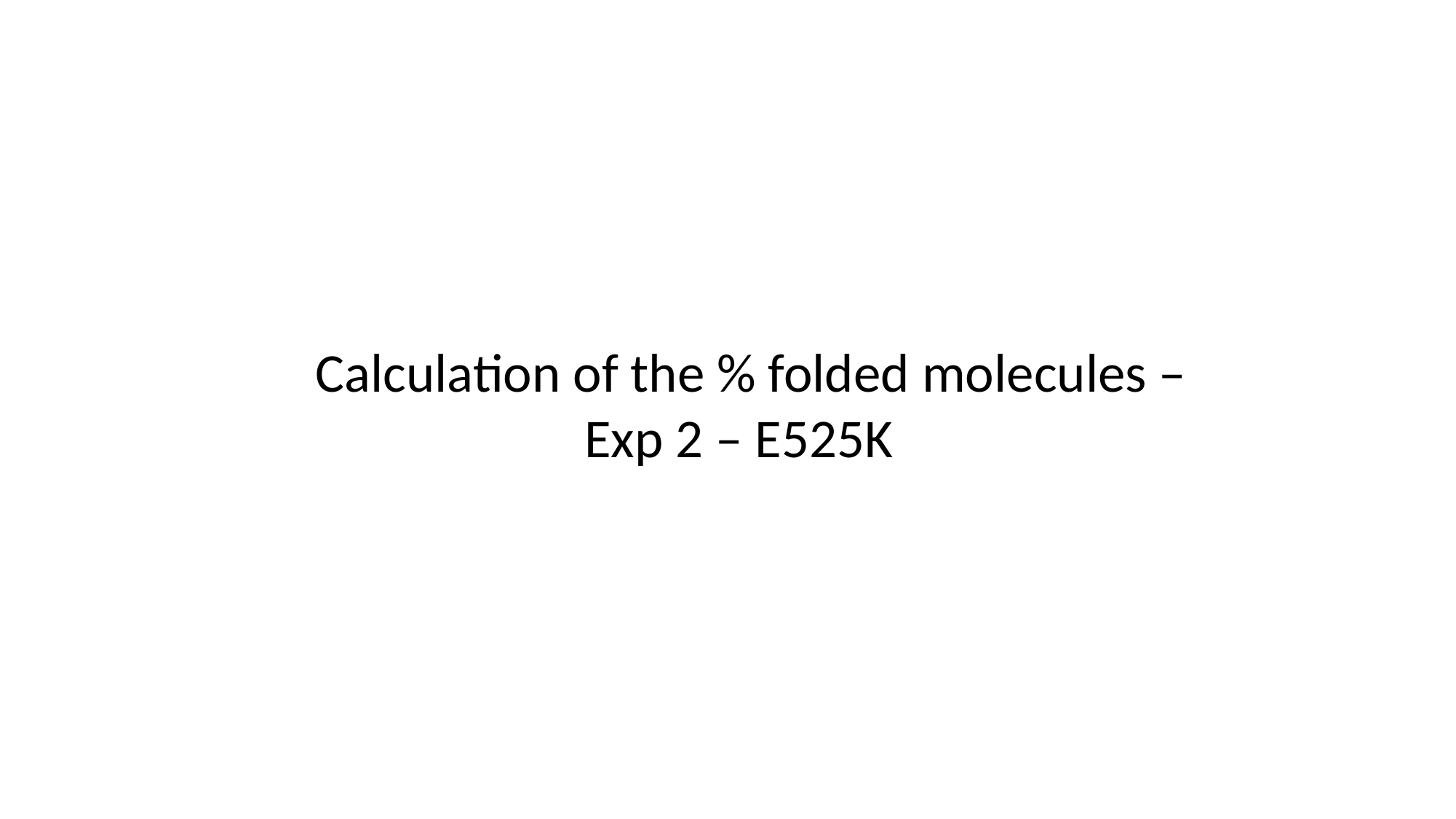

Calculation of the % folded molecules – Exp 2 – E525K

## Slide 2
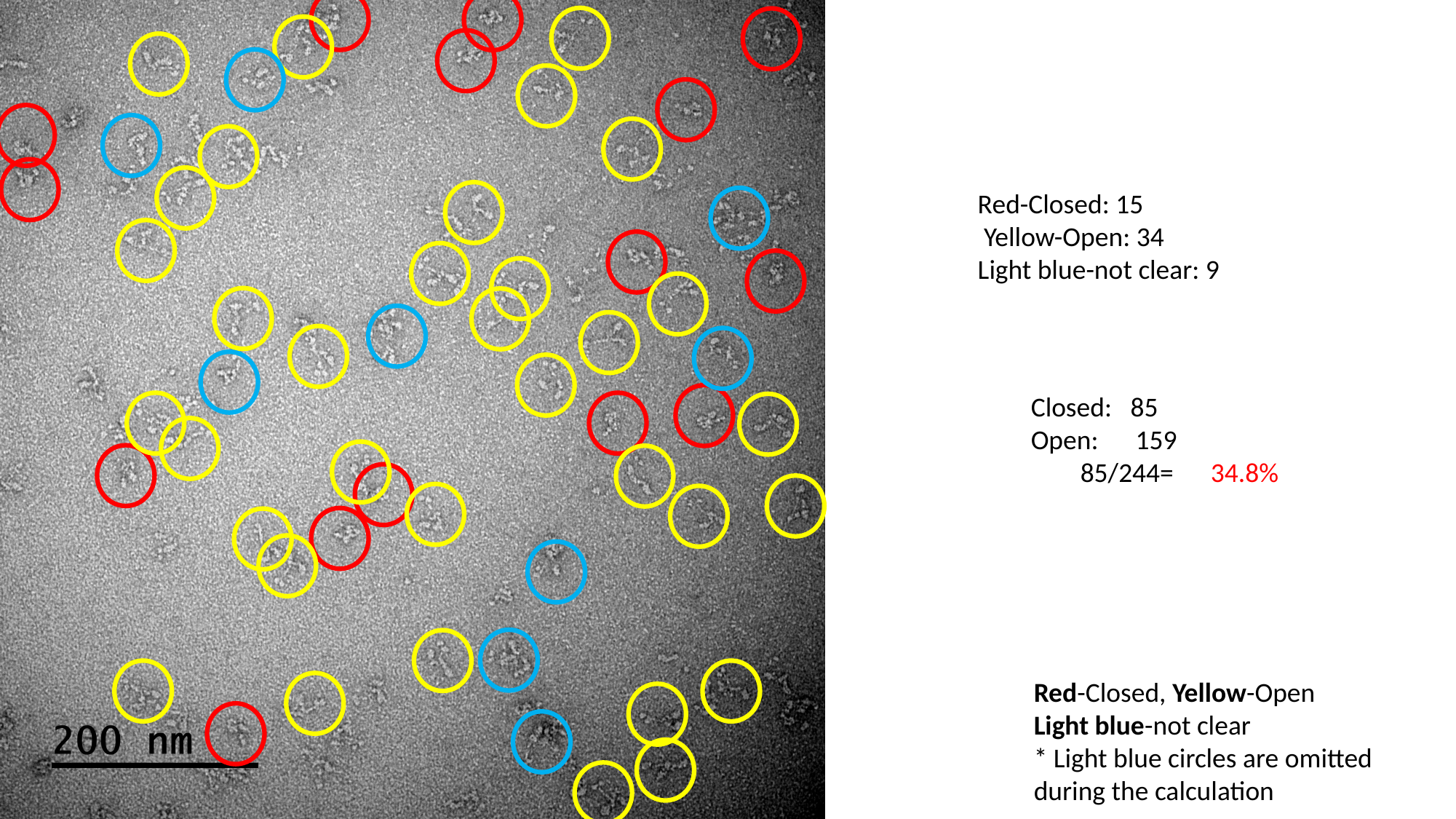

Red-Closed: 15
 Yellow-Open: 34
Light blue-not clear: 9
Closed: 85
Open: 159
 85/244= 34.8%
Red-Closed, Yellow-Open
Light blue-not clear
* Light blue circles are omitted during the calculation

## Slide 3
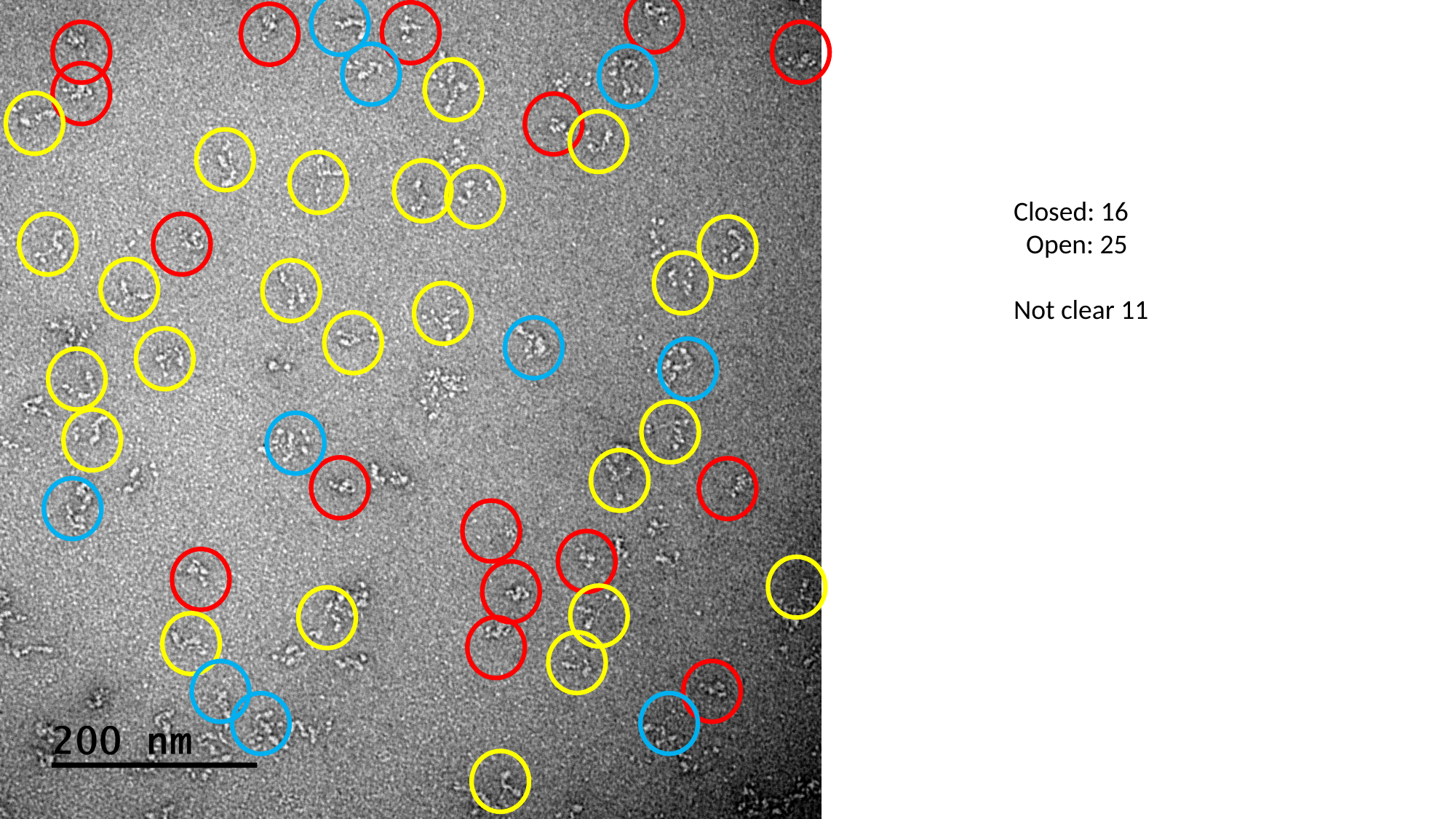

Closed: 16
 Open: 25
Not clear 11

## Slide 4
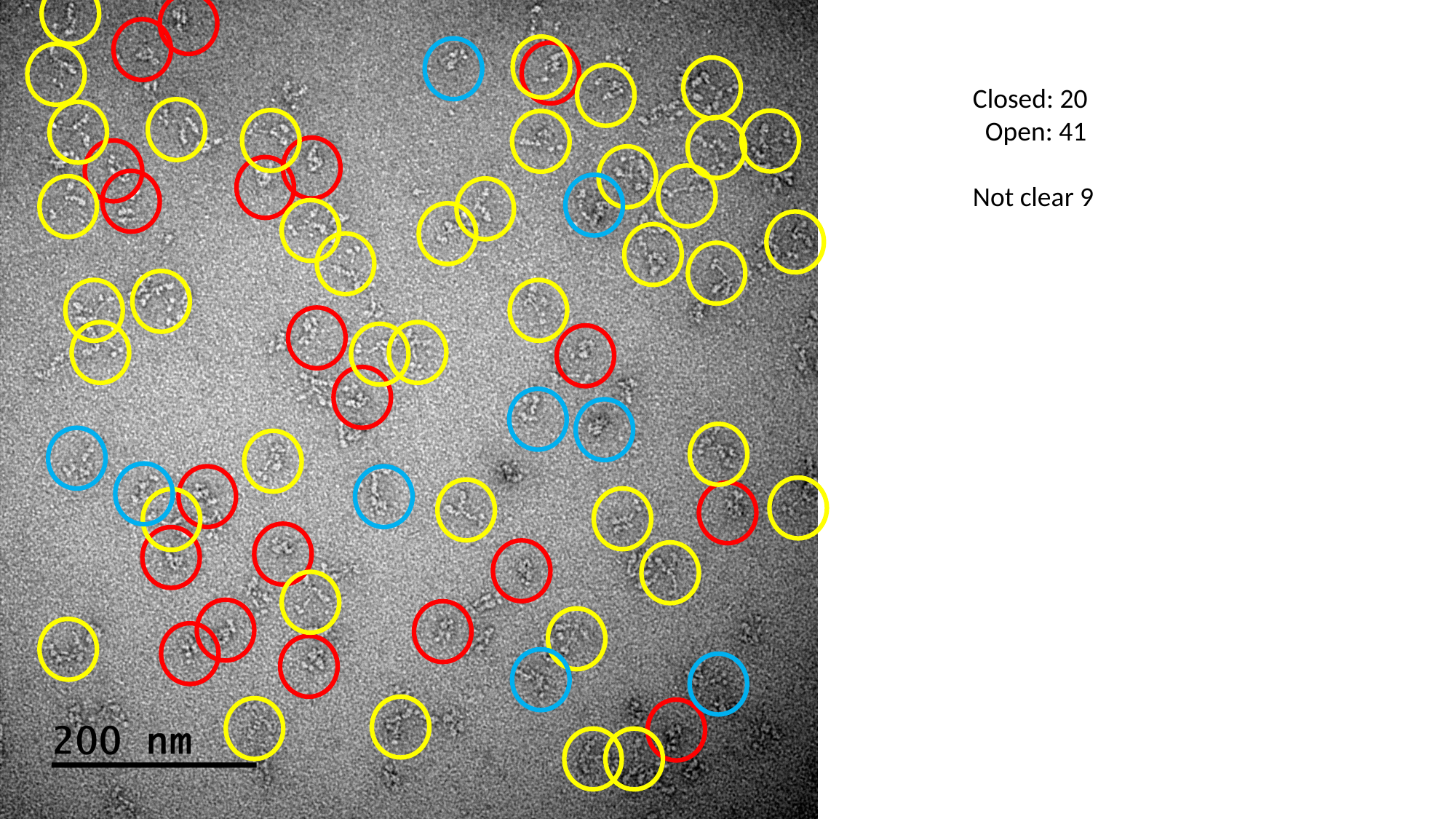

Closed: 20
 Open: 41
Not clear 9

## Slide 5
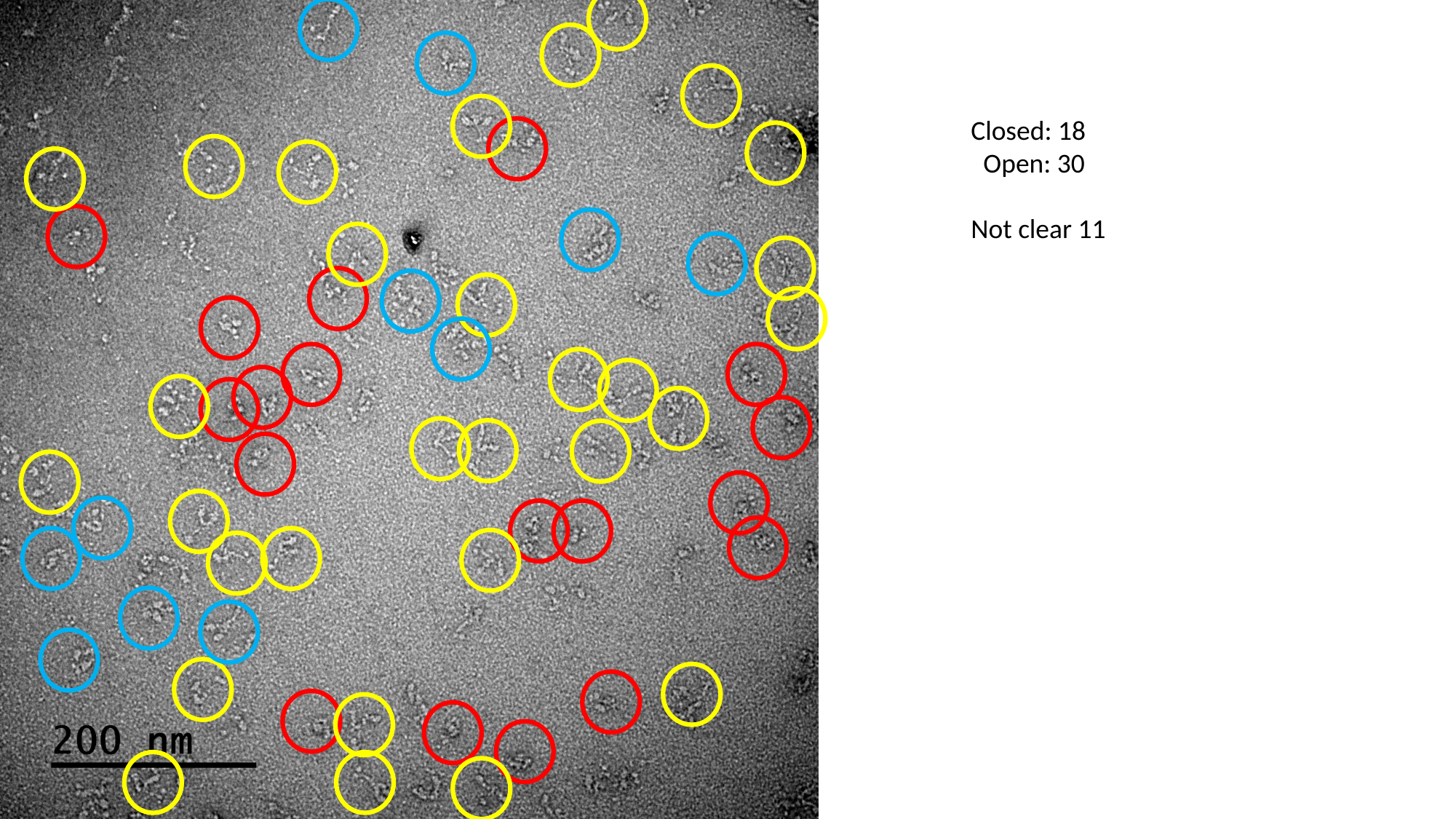

Closed: 18
 Open: 30
Not clear 11

## Slide 6
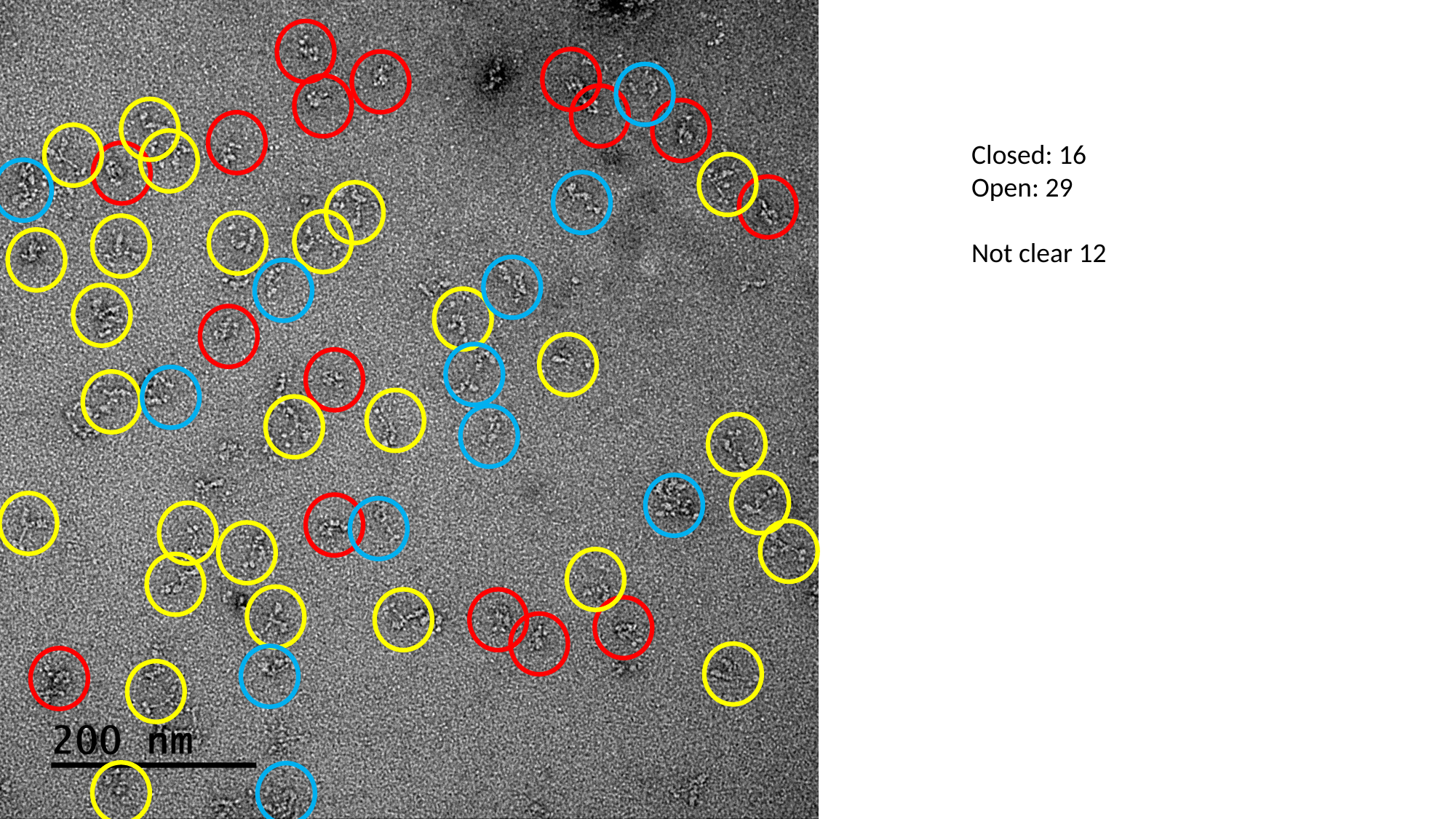

Closed: 16
Open: 29
Not clear 12
