# Supplementary material for: Dilated cardiomyopathy mutation E525K in human beta-cardiac myosin stabilizes the interacting-heads motif and super-relaxed state of myosin
Source: eLife. 2022 Nov 24;11:e77415. doi: 10.7554/eLife.77415 (PMC9691020; doi:10.7554/eLife.77415)
Supplement: Figure 6—source data 1. [file elife-77415-fig6-data1.zip › 2nd prep_05-10-2022/WT/WT.pptx]

## Slide 1
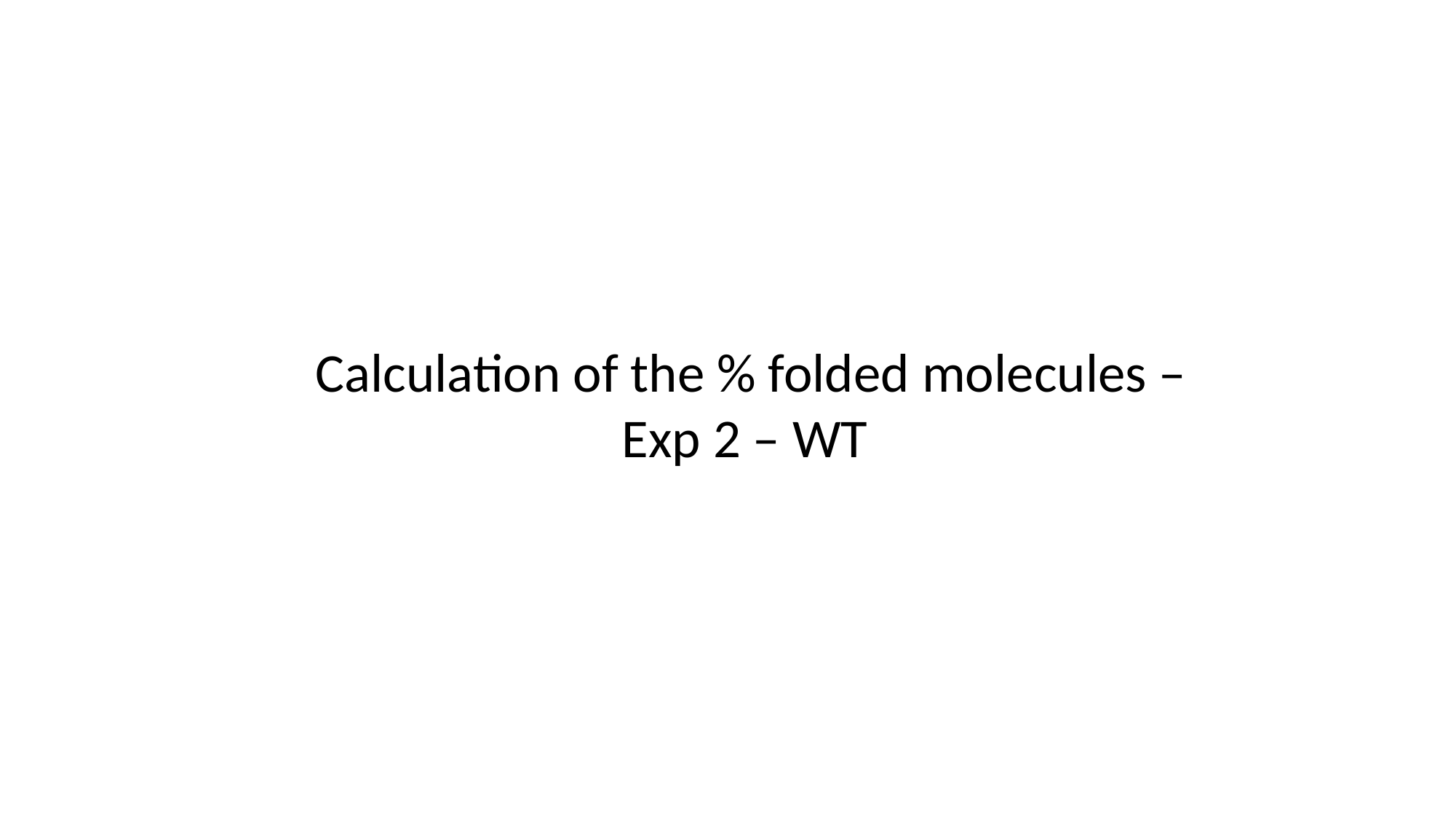

Calculation of the % folded molecules – Exp 2 – WT

## Slide 2
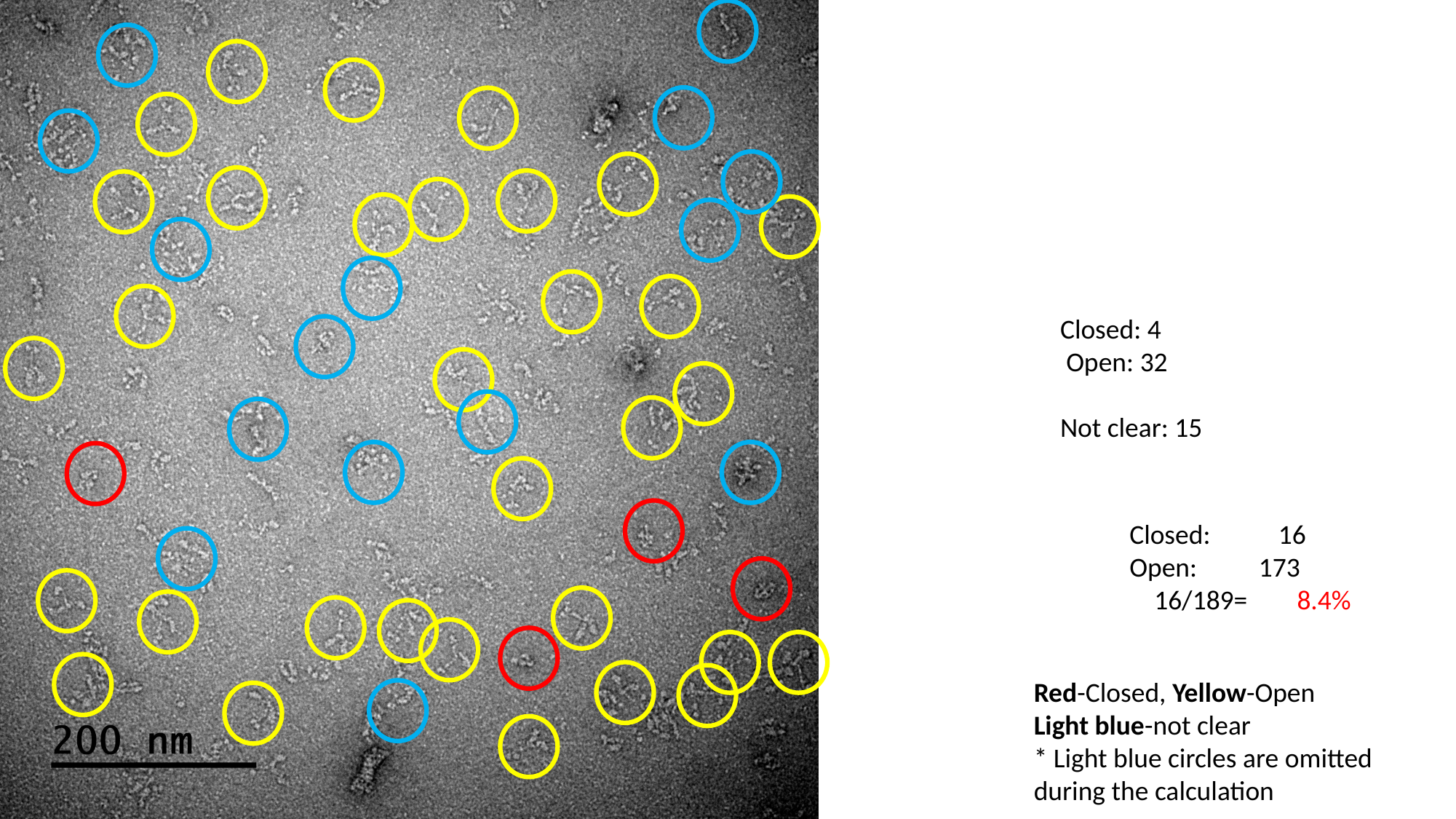

Closed: 4
 Open: 32
Not clear: 15
Closed: 16
Open: 173
 16/189= 8.4%
Red-Closed, Yellow-Open
Light blue-not clear
* Light blue circles are omitted during the calculation

## Slide 3
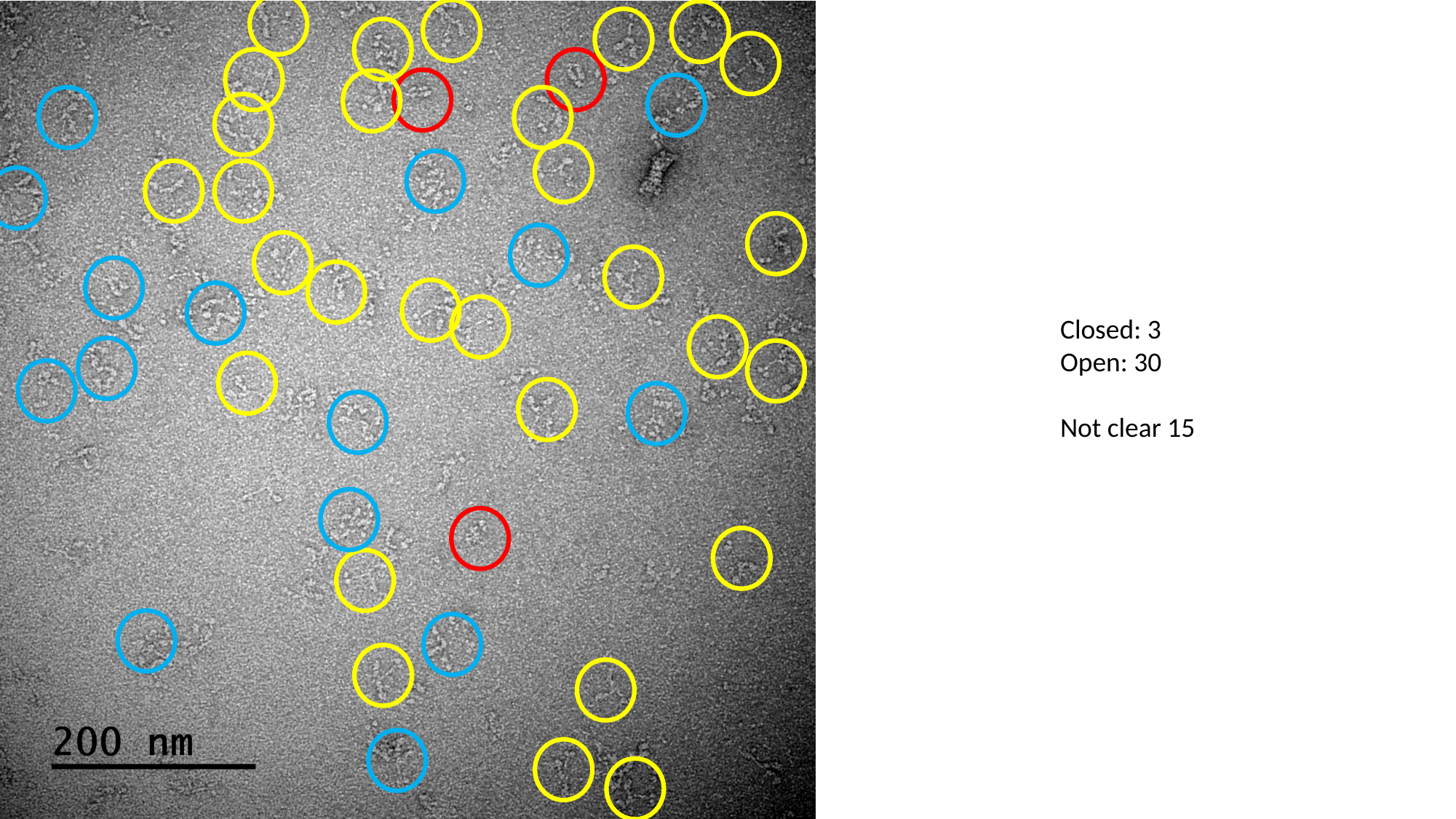

Closed: 3
Open: 30
Not clear 15

## Slide 4
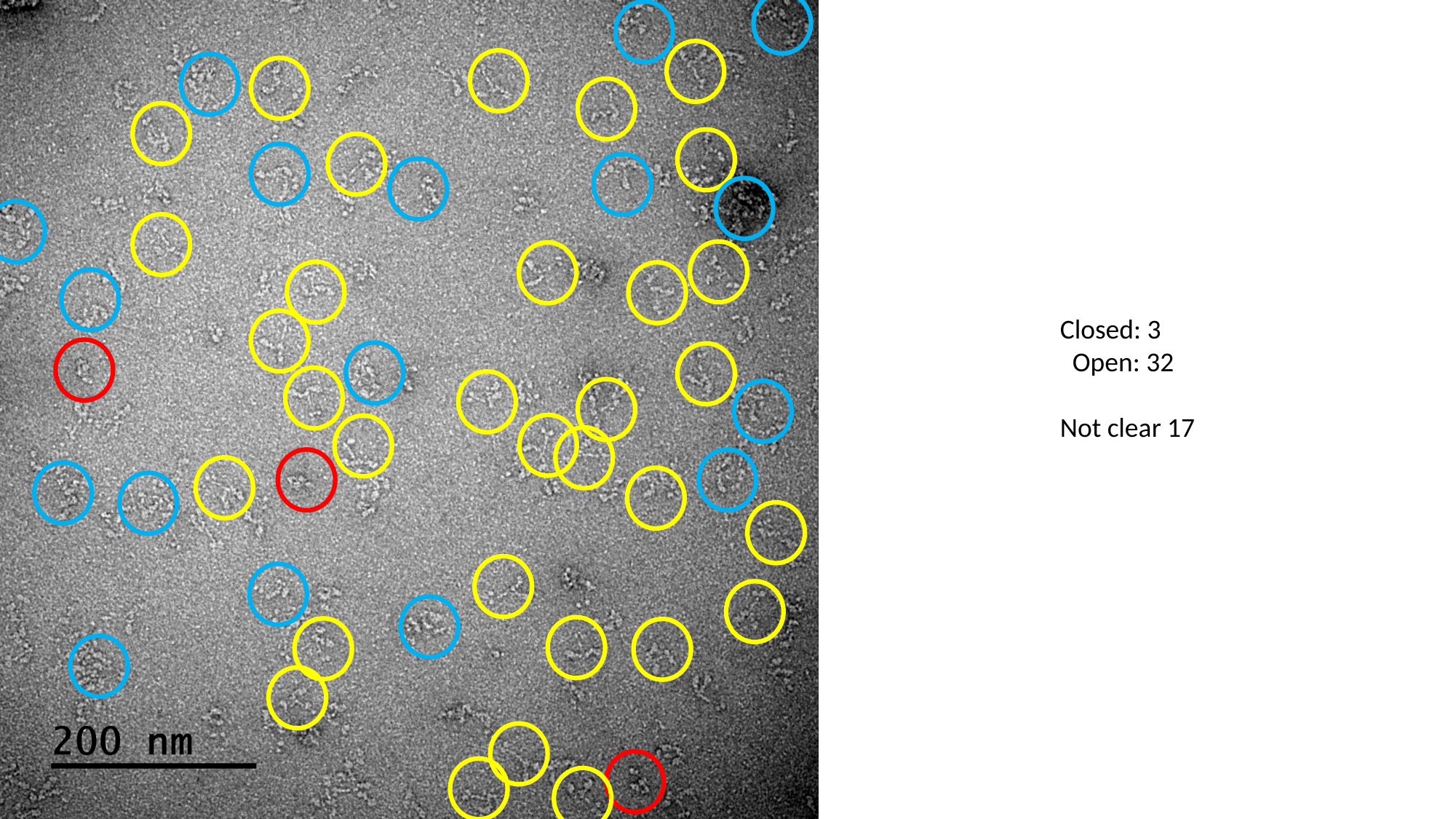

Closed: 3
 Open: 32
Not clear 17

## Slide 5
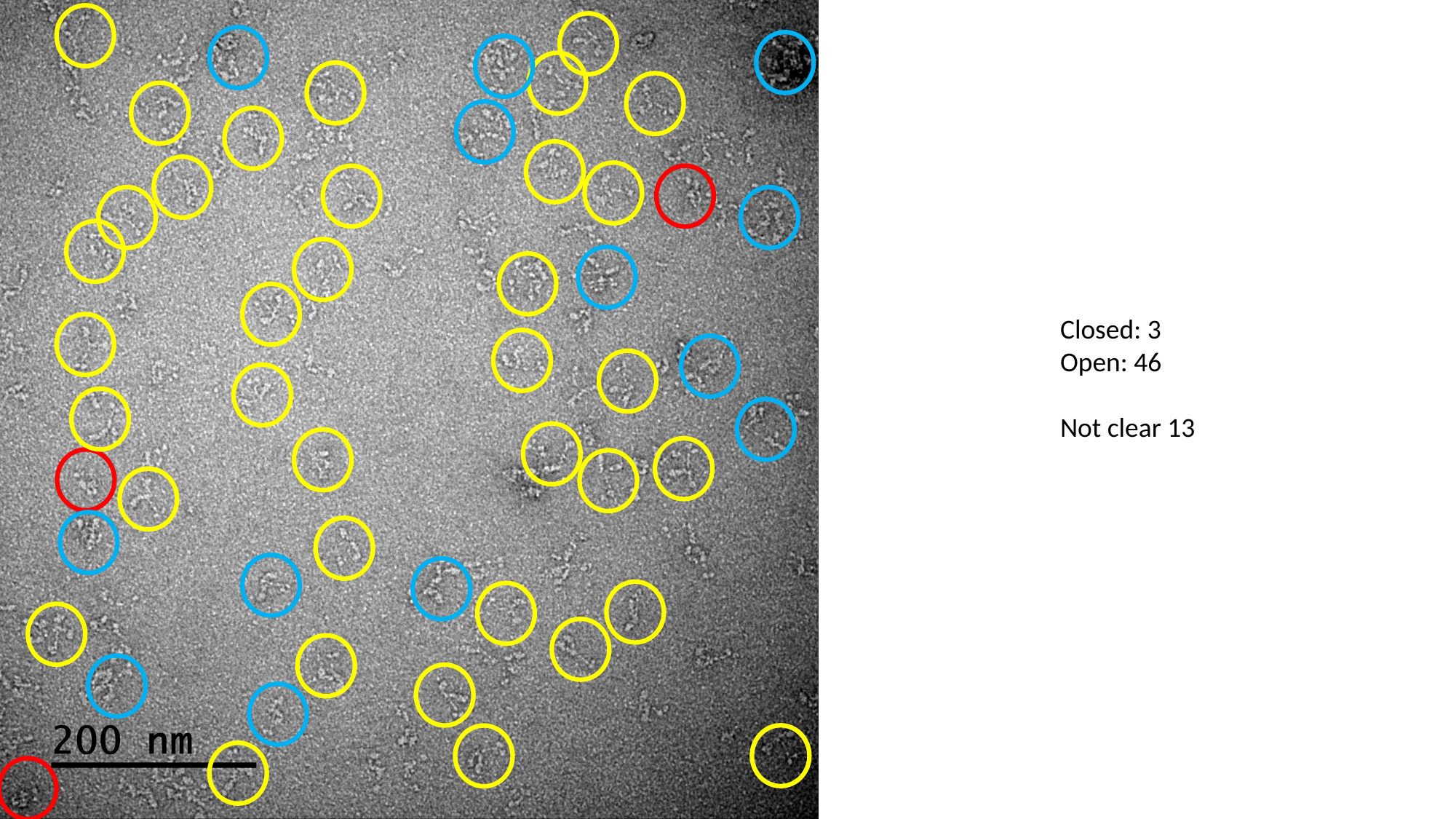

Closed: 3
Open: 46
Not clear 13

## Slide 6
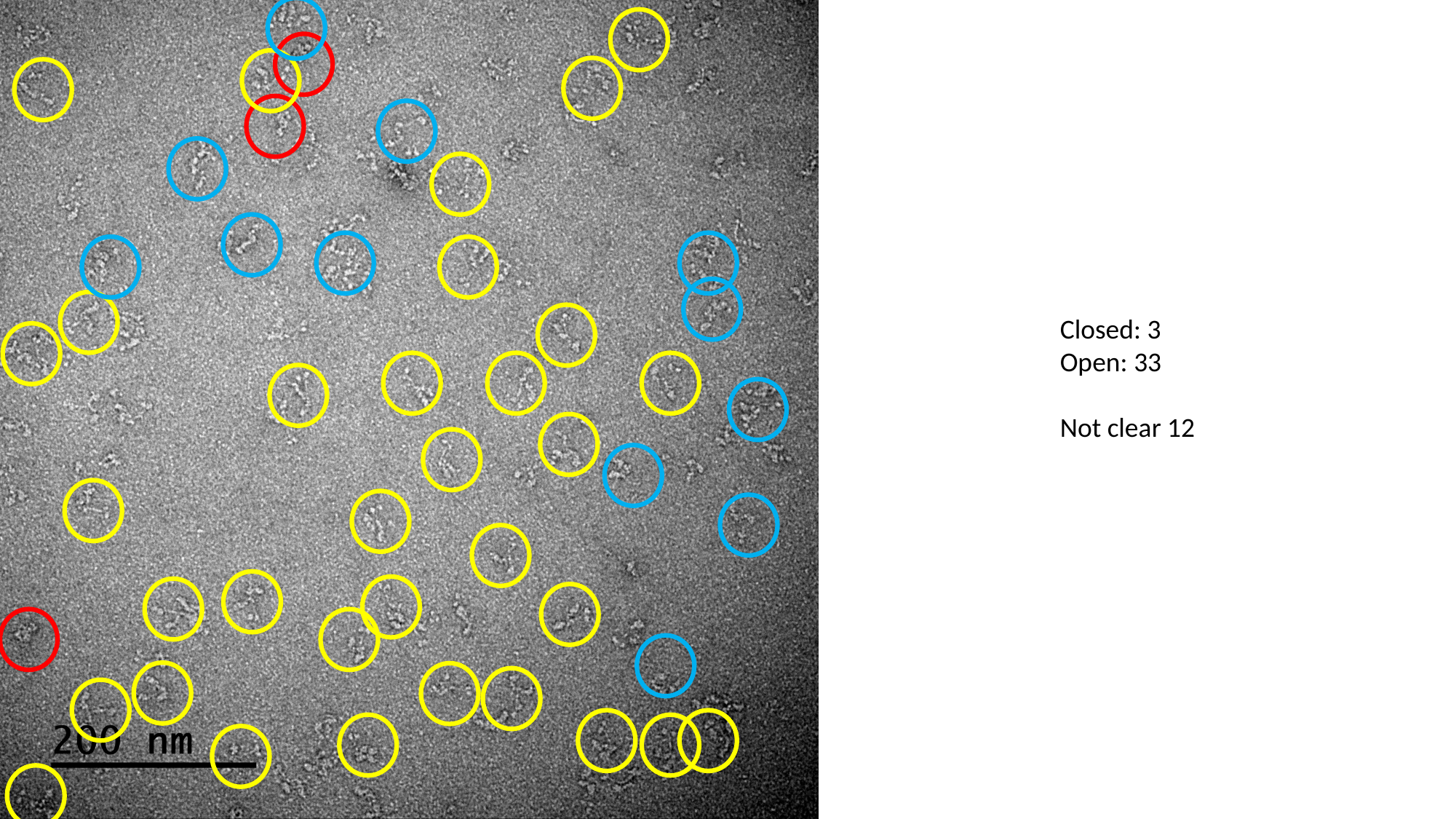

Closed: 3
Open: 33
Not clear 12
