# Supplementary material for: Dilated cardiomyopathy mutation E525K in human beta-cardiac myosin stabilizes the interacting-heads motif and super-relaxed state of myosin
Source: eLife. 2022 Nov 24;11:e77415. doi: 10.7554/eLife.77415 (PMC9691020; doi:10.7554/eLife.77415)
Supplement: Figure 6—source data 1. [file elife-77415-fig6-data1.zip › 3rd prep_05-30-2022/WT/WT.pptx]

## Slide 1
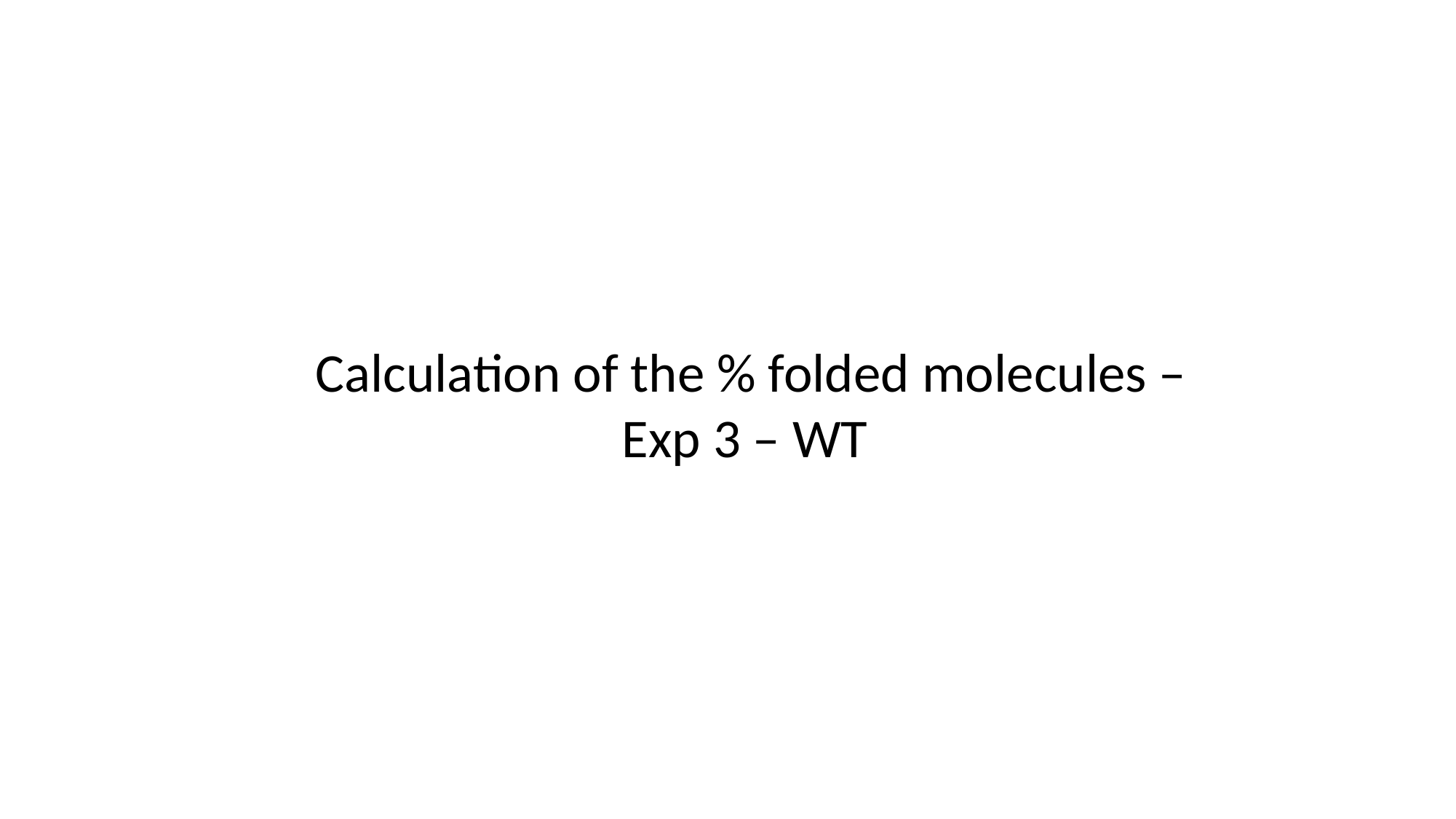

Calculation of the % folded molecules – Exp 3 – WT

## Slide 2
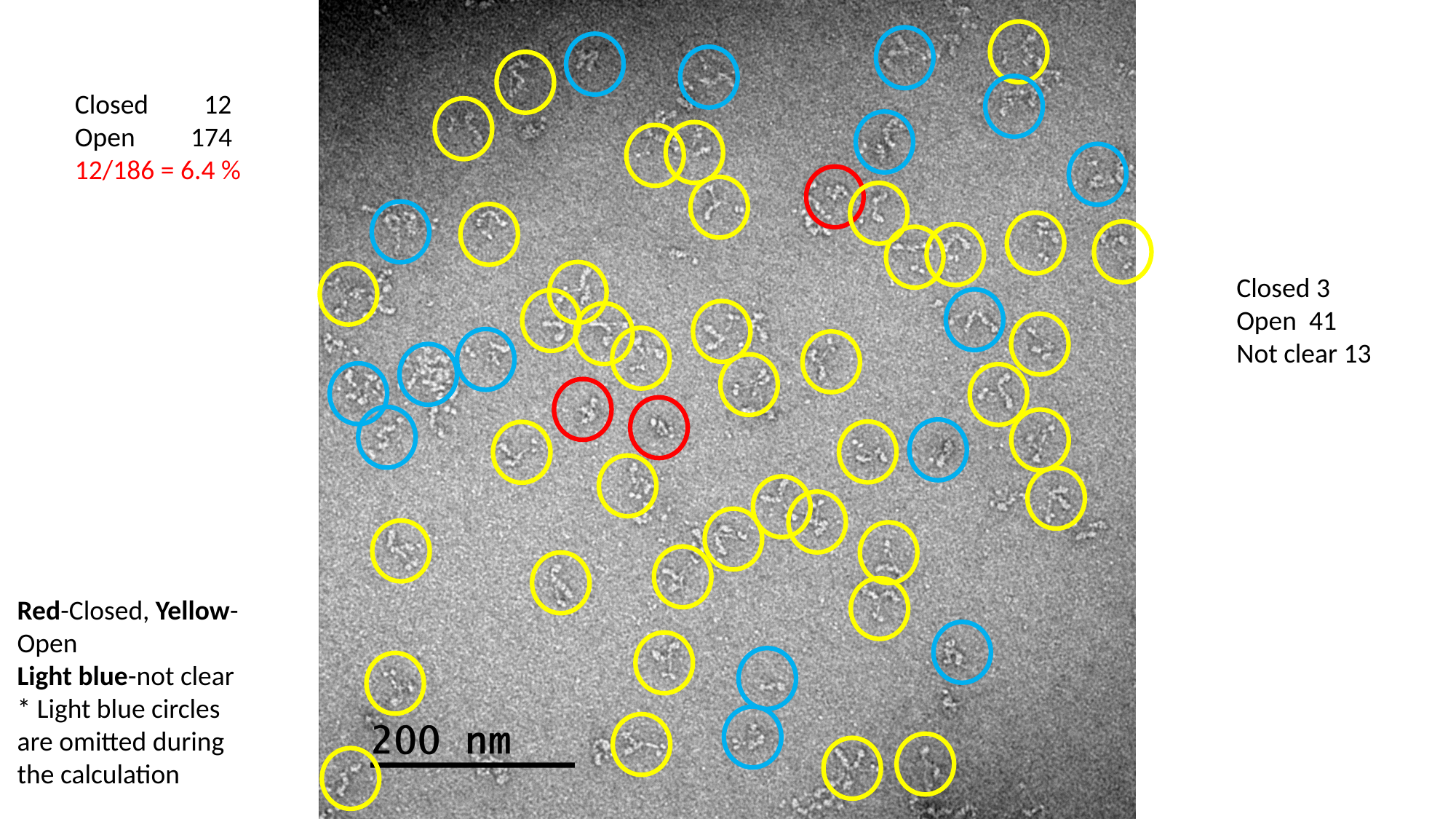

Closed 12
Open 174
12/186 = 6.4 %
Closed 3
Open 41
Not clear 13
Red-Closed, Yellow-Open
Light blue-not clear
* Light blue circles are omitted during the calculation

## Slide 3
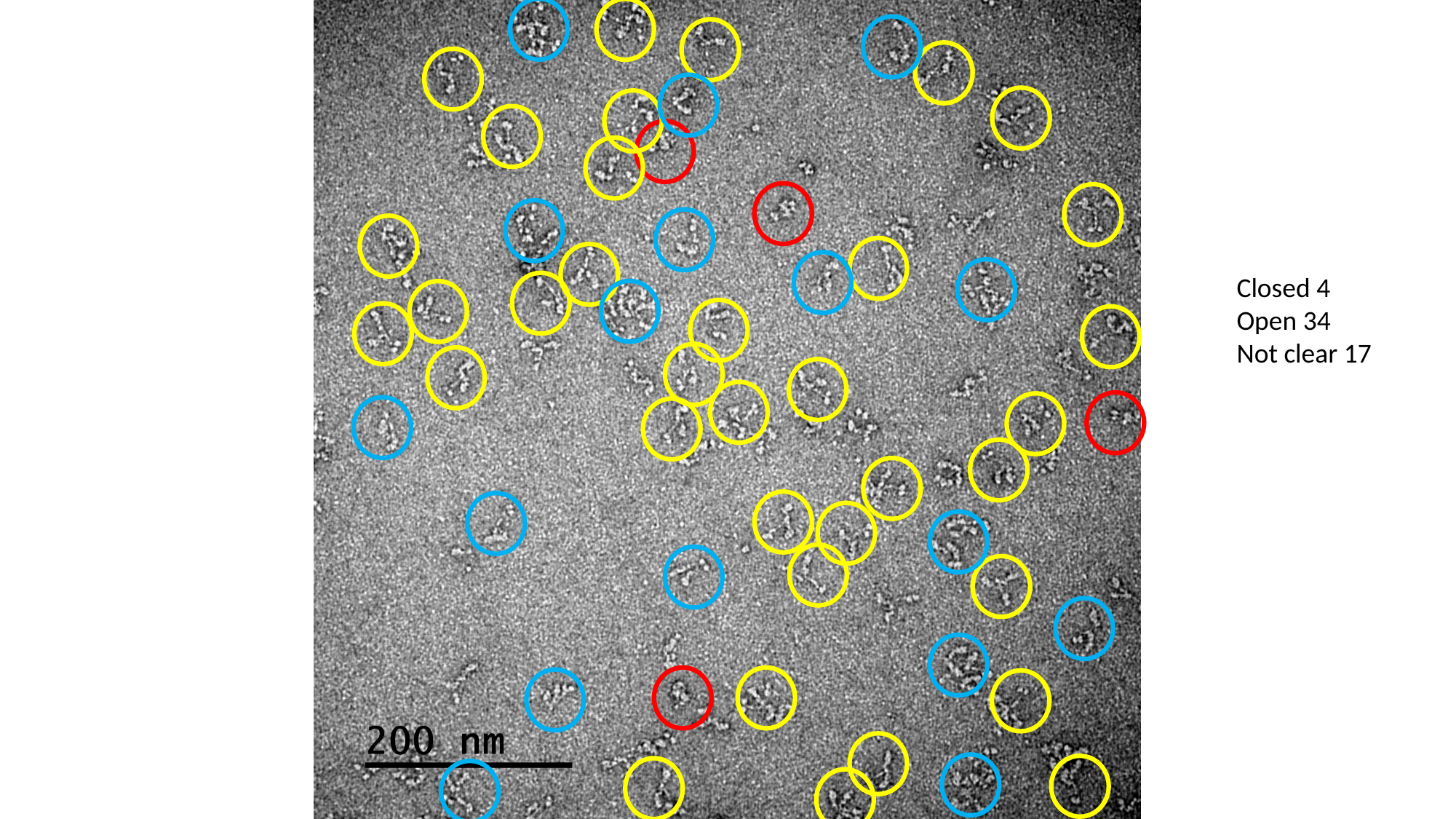

Closed 4
Open 34
Not clear 17

## Slide 4
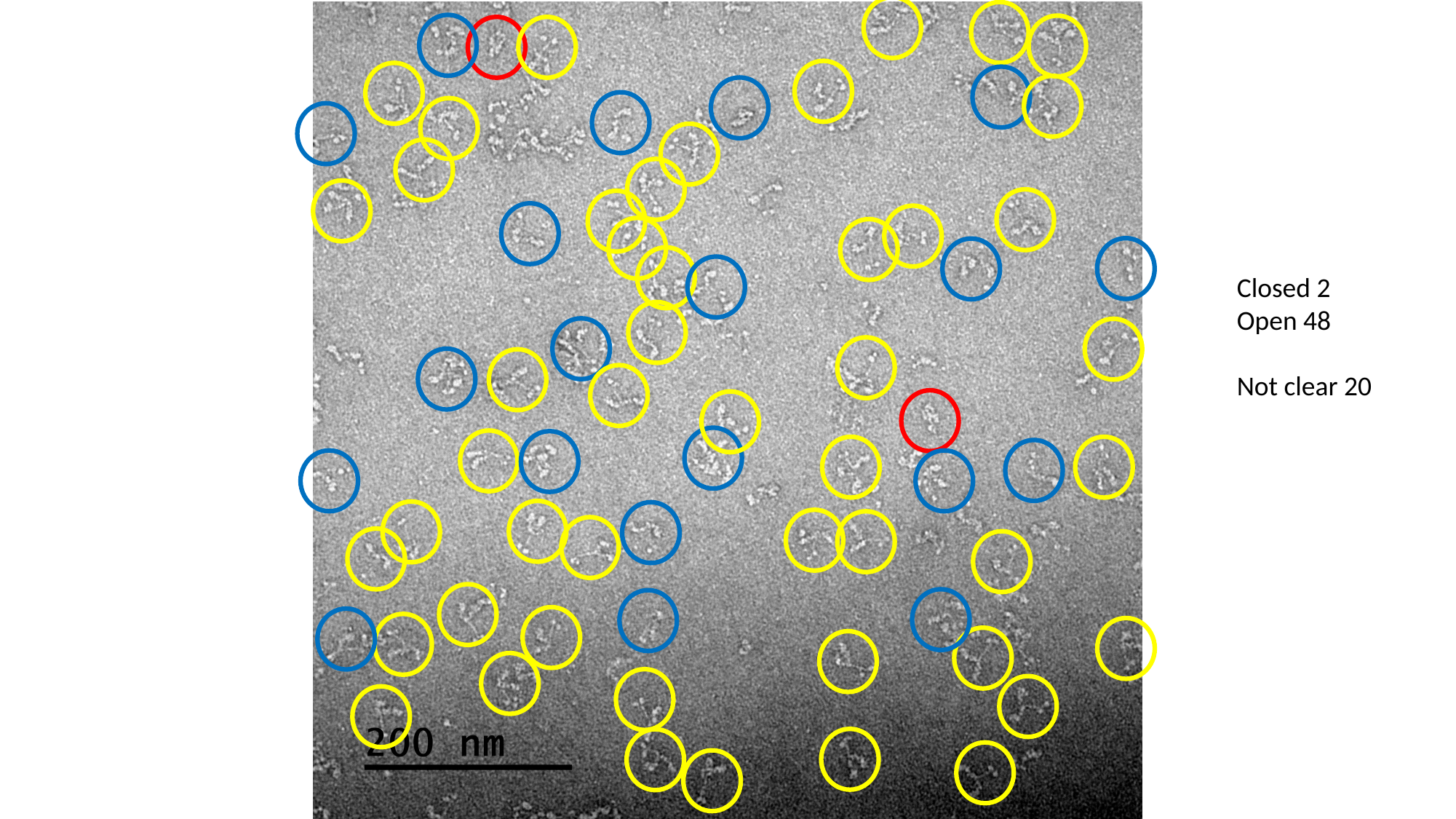

Closed 2
Open 48
Not clear 20

## Slide 5
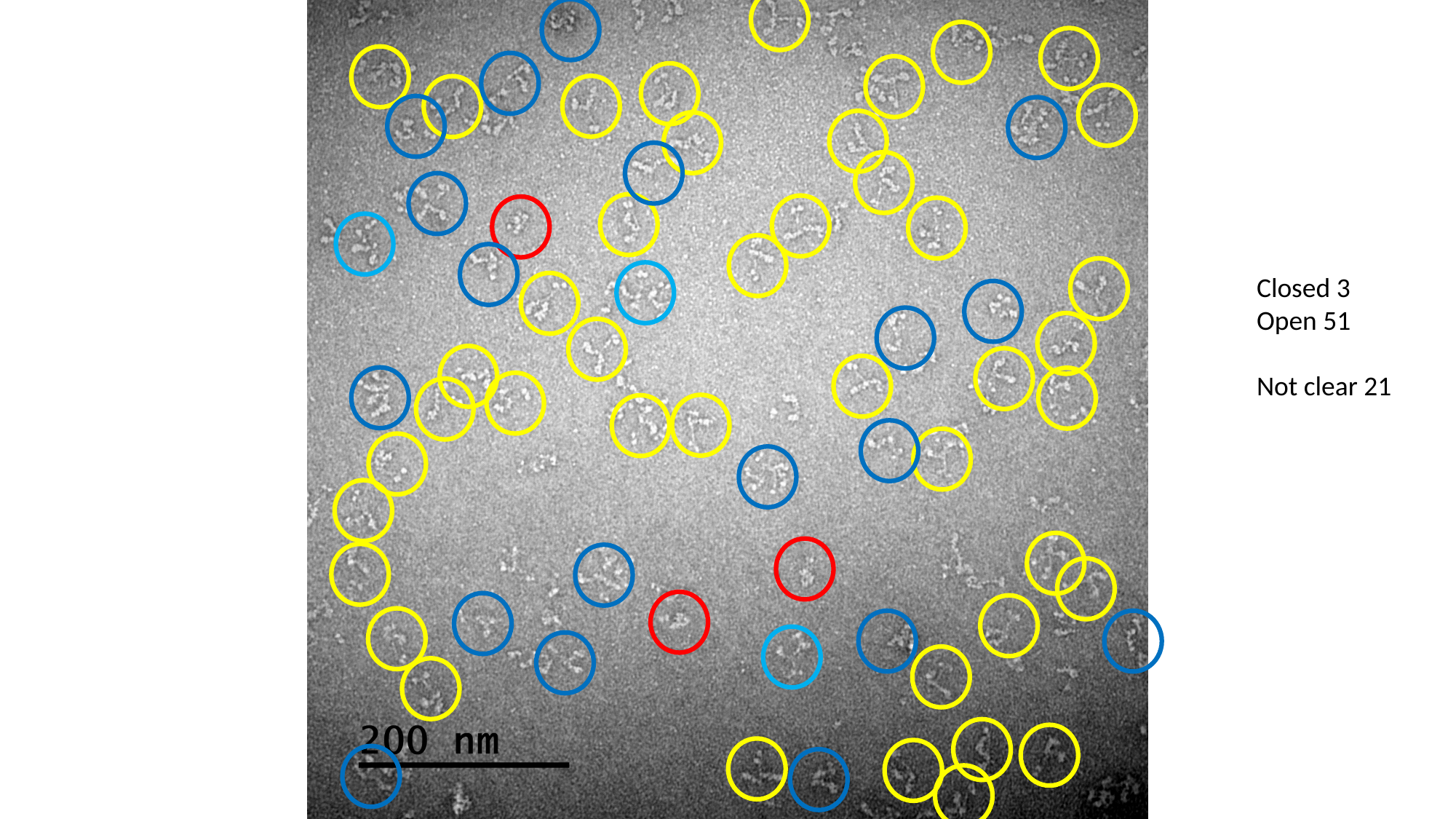

Closed 3
Open 51
Not clear 21
